# Impact of enzyme replacement therapy and migalastat on left atrial strain and cardiomyopathy in patients with Fabry disease

**DOI:** 10.3389/fcvm.2023.1223635

**Published:** 2023-11-02

**Authors:** Christian Pogoda, Stefan-Martin Brand, Thomas Duning, Antje Schmidt-Pogoda, Jürgen Sindermann, Malte Lenders, Eva Brand

**Affiliations:** ^1^ Department of Cardiology I—Coronary and Peripheral Vascular Disease, Heart Failure, and Interdisciplinary Fabry Center (IFAZ), University Hospital Münster, Münster, Germany; ^2^ Institute of Sports Medicine, Molecular Genetics of Cardiovascular Disease, and Interdisciplinary Fabry Center (IFAZ), University Hospital Münster, Münster, Germany; ^3^ Department of Neurology, and Interdisciplinary Fabry Center (IFAZ), University Hospital Münster, Münster, Germany; ^4^ Department of Neurology, Klinikum Bremen-Ost, Bremen, Germany; ^5^ Interdisciplinary Heart Failure Section, University Hospital Münster, Münster, Germany; ^6^ Department of Internal Medicine D, and Interdisciplinary Fabry Center (IFAZ), University Hospital Münster, Münster, Germany

**Keywords:** cardiomyopathy, Fabry disease, migalastat, enzyme replacement therapy, left atrial strain, speckle tracking

## Abstract

**Aims:**

Cardiomyopathy in Fabry disease (FD) is a major determinant of morbidity and mortality. This study investigates the effects of FD-specific treatment using enzyme replacement therapy (ERT) and chaperone therapy on left atrial (LA) function using two-dimensional speckle tracking echocardiography (2DSTE).

**Methods and results:**

In this prospective observational single-center study, 20 FD patients [10 (50%) females] treated with migalastat, 48 FD patients [24 (50%) females] treated with ERT (agalsidase-alfa and agalsidase-beta), and 30 untreated FD patients (all females) as controls were analyzed. The mean follow-up time ranged from 26 to 81 months. 2DSTE was performed for left ventricle strain, right ventricle strain, and LA strain (LAS). FD-specific treated patients presented with increased left ventricular mass index (LVMi) and higher frequency of left ventricular hypertrophy at baseline, whereas untreated control patients showed normal baseline values. FD-specific treated (including migalastat and ERT) patients showed stabilization of LAS over time (*p* > 0.05). LVMi was also stable in treated FD patients during observation (*p* > 0.05).

**Conclusion:**

In patients with FD, treated with either ERT or chaperone therapy, LAS values measured by echocardiographic speckle tracking were stable over time, pointing toward disease stabilization.

## 1. Introduction

In Fabry disease (FD), cardiac involvement is a major cause of premature death (1, 2). FD is a rare X-linked lysosomal storage multisystem disease because of the deficiency of the enzyme *α*-galactosidase A (AGAL). An ongoing cellular accumulation of globotriaosylceramide (Gb_3_) results in a progressive structural heart disease starting in childhood (3). The main aspect of developing cardiomyopathy in FD is left ventricular (LV) hypertrophy (LVH) (4). Life-long treatment with intravenous enzyme replacement therapy [ERT; agalsidase-alfa (Takeda), agalsidase-beta (Sanofi)] or oral chaperone therapy [migalastat (Amicus Therapeutics)] represent FD-specific treatments, which can counteract premature death and attenuate symptoms/organ manifestations (5). However, if FD-specific therapy is initiated later, disease progression can only be delayed, and existing organ damage, such as FD cardiomyopathy, may become irreversible, suggesting a point of no return (6, 7). If left untreated, FD patients show reduced life expectancy in both males (58.2 vs. 74.7 years) and females (75.4 vs. 80.0 years) (8). Thus, it is important to diagnose FD early, determine the degree of cardiac involvement, initiate FD-specific treatment (if indicated), and monitor response to therapy. The initiation of FD-specific therapy can be triggered not only by cardiomyopathy but also by a range of organ damages, including chronic kidney disease and neurological symptoms, as well as preemptively in “classic” male patients, highlighting the diverse clinical indications that underlie treatment decisions (9).

Echocardiography strain imaging by tissue Doppler or speckle tracking allows early detection (10) and precise follow-up of FD cardiomyopathy (11). Prior to the development of LVH, a strain pattern with LV posterolateral strain impairment for the LV is characteristic of FD (12). In addition to strain analyses of the LV, 2D-speckle tracking (2DSTE) of the left atrium (LA) has shown prognostic capabilities in cardiomyopathies with hypertrophic phenotype in recent years, such as predicting a negative disease course in hypertrophic cardiomyopathy (HCM) (13). Furthermore, the prognostic value of LA strain (LAS) has been demonstrated for atrial fibrillation in AL-amyloidosis (14), as well as atrial fibrillation and stroke in the general population (15).

For FD, a worse symptomatic status (16) is associated with decreasing LAS. Therefore, LAS analyses seem to be an appropriate method to evaluate the effect of therapy on FD cardiomyopathy.

Although FD cardiomyopathy is the main cause of death in FD, literature addressing the effects of FD-specific treatment on FD cardiomyopathy and, particularly, the LA is scarce. Hence, our study aims to describe the changes in the cardiac structure, focusing on the LA, in a large cohort of FD patients treated with either ERT or chaperone therapy. To this end, we used 2DSTE, ideal for early detection and precise follow-up of FD cardiomyopathy.

## 2. Materials and methods

### 2.1. Study design

A total of 98 patients (64 females) with genetically confirmed FD were recruited at the Interdisciplinary Fabry Center Muenster (IFAZ) and followed in a prospective observational study (Supplementary Figure S1). The inclusion criteria were (a) age ≥16 years and a genetically confirmed *GLA* mutation, (b) therapy-naïve status at baseline (controls) or ≥12 months of ERT with recommended doses of agalsidase-alfa (0.2 mg/kg e.o.w.) or agalsidase-beta (1.0 mg/kg e.o.w.) before inclusion, (c) written informed consent for study participation as well as molecular analysis and publication, and (d) a follow-up period of ≥18 months (untreated or migalastat- or ERT-treated). All investigations were performed after the approval of the Medical Association of Westfalian-Lippe and the Ethical Committee of the Medical Faculty of the University of Muenster (project nos.: 2011–347-f, date of report: 07 July 2011). Patients with genetic variances of unknown significance (GVUS) [p.A143T, p.S126G, p.R118C] received FD-specific treatment if at least one end-organ damage was detected, justifying FD-specific treatment (9).

All patients underwent echocardiography at baseline and each follow-up according to current recommendations (17). The ultrasound systems used were GE Vivid 7, GE E95, Philips IE 33, and Philips Epiq 7. Measurements and LV mass index (LVMi) calculations adhered to the Chamber Quantification guidelines (18) and were performed offline and blinded by a single experienced investigator using TOMTEC Image-Com (TOMTEC Imaging Systems GmbH, Unterschleissheim, Germany, version 11.0.5.10.1, AutoStrainCap2 2.1.0.494368).

LV mass (LVM) and LV mass index (LVMi) were calculated using the Devereux formula (19) by LV cavity dimension and wall thickness at end-diastole. The presence of LVH was defined as an LVMi above the appropriate reference ranges [LVMi, reference ranges 43–95 (female) and 49–115 (male) g/m^2^] (20).

2DSTE was performed for LV, right ventricular (RV), and LAS following recent recommendations (21–23) using TOMTEC AutoStrainCap2. For LV, the endocardium, which is the innermost layer, was manually delineated, after which the software autonomously traced the regions of interest (ROIs). The examiner manually fine-tuned the tracking as required. The LV strain was measured at the peak systolic longitudinal strain and with endocardial tracing. Measurements were undertaken from apical views, e.g., apical four-, three-, and two-chamber views, and integrated into the 17-segment model. In assessing LAS, the boundaries of the LA were manually marked, succeeded by software-aided tracking. Adjustments of ROI and tracking were made manually when necessary. The ECG R wave served as the trigger for end-diastole, with the addition of mitral valve closure used as a reference in cases of uncertainty. Data were aggregated from apical four- and two-chamber views for an average value. RV longitudinal strains were measured using the four-chamber view. We employed TOMTEC for RV wall detection, and ROIs were manually modified when necessary. The cardiac cycle was defined using an ECG trigger, and when in doubt, it was corrected by observing the closure of the tricuspid valve.

To measure consistency across observers, 10 sets of strain measurements for LV and LA were performed. Our primary examiner and an additional observer independently conducted these measurements, facilitating the computation of *R*^2^ for intraobserver variability (Supplementary Figure S2). For the assessment of interobserver variability, the primary examiner performed 10 random measurements twice. *R*^2^ was subsequently determined to be >0.5 (Supplementary Figure S3), which is sufficient.

The estimated glomerular filtration rate (eGFR) was quantified using the Chronic Kidney Disease-Epidemiology Collaboration (CKD-EPI)-based equation based on serum creatinine (eGFRcreat) (24). Albuminuria was defined as an albumin–creatinine ratio (ACR) >30 mg albumin per gram of creatinine from spot urine.

The plasma lyso-Gb_3_ levels were measured in one laboratory (Centogene, Rostock, Germany).

### 2.2. Data analyses and statistics

Data are presented as mean ± SD, mean (95% confidence intervals), median (min–max), or number (percentage) if not stated otherwise. Baseline and follow-up values were compared using paired tests (Student’s or Wilcoxon), where appropriate. The differences between groups were tested via one-way ANOVA between females or Student’s *t*-test between males for continuous variables. For categorical data, the chi-squared or Fisher’s exact tests were used. All differences were tested two-sided. *p*-values of <0.05 were considered statistically significant. Data were analyzed using SAS version 9.4 (SAS Institute, Cary, NC, USA) and GraphPad PRISM V5.0 software (GraphPad Software, La Jolla, CA). Some figures were created using BioRender.

## 3. Results

### 3.1. Baseline characteristics of the study population

In total, 20 patients [10 (50%) females] receiving migalastat, 48 patients [24 (50%) females] receiving ERT, and 30 untreated FD (all females) controls were prospectively analyzed (Supplementary Figure S1). Table 1 presents the baseline characteristics. While 40% of patients receiving migalastat were previously treated with ERT, all except one female patient in the ERT group were ERT-naïve at baseline. Females treated with migalastat were significantly older than untreated (controls) and ERT-treated females (*p* = 0.0002). A comparable trend was observed for male patients (*p* = 0.0836). The longest follow-ups were available for female and male patients treated with ERT (both *p* = 0.0001, Table 1). Refer to Supplementary Table S1 for differences between females and males within the groups. Kidney function (eGFR and albuminuria) was comparable between all groups. As a marker of disease burden, plasma lyso-Gb_3_ had the highest value in ERT-treated patients (females: *p* = 0.0441; males: *p* = 0.0314). Male patients presented with the highest plasma lyso-Gb_3_ values (migalastat group: *p* = 0.0364; ERT group: *p* = 0.0109). Females receiving ERT showed the highest septum thickness (*p* = 0.0030), while septum thickness between males was comparable. In addition, LVMi was the highest in females receiving ERT (*p* = 0.0173). As expected, patients with non-sense mutations were only present in the ERT group and the untreated controls but not in migalastat-treated patients (Table 1). The late-onset mutation p.N215S was most present in the migalastat-treated males (*p* = 0.0198).

**Table 1:** Baseline characteristics of the study cohort.

Group	Females				Males		
	Untreated (*n* = 30)	Migalastat-treated (*n* = 10)	ERT-treated (*n* = 24)	*p*-value^a^	Migalastat-treated (*n* = 10)	ERT-treated (*n* = 24)	*p*-value^b^
Age (years)	28 (16–68)	62 (44–72)^c^,***	48 (25–71)^c^,*	**0**.**0002**	52 (16–68)	36 (18–65)	0.0836
Mean follow-up (months)	34 ± 12	28 ± 10	75 ± 40^c^,***	**0**.**0001**	26 ± 6	81 ± 39	**0**.**0001**
Pre-treated with ERT, *n* (%)	na	4 (40.0)	1 (4.2)	**0**.**0001**	4 (40.0)	0 (0.0)	**0**.**0045**
Angioceratoma, *n* (%)	4 (13.8)	2 (20.0)	9 (39.1)	0.1000	1 (10.0)	15 (65.2)	**0**.**0066**
Edema, *n* (%)	0 (0.0)	1 (10.0)	0 (0.0)	0.0612	2 (20.0)	5 (20.8)	0.9999
Diarrhea, *n* (%)	8 (25.8)	4 (44.4)	6 (25.0)	0.5005	0 (0.0)	8 (36.4)	0.0735
Abdominal pain, *n* (%)	10 (33.3)	5 (50)	6 (25.0)	0.3647	4 (40.0)	7 (30.4)	0.6960
FD-specific pain, *n* (%)	14 (45.2)	6 (60.0)	16 (66.7)	0.2680	6 (60.0)	14 (58.3)	0.9999
Hypohidrosis, *n* (%)	5 (16.1)	2 (20.0)	8 (34.8)	0.2674	2 (20.0)	13 (54.2)	0.1285
Creatinine (mg/dl)	0.67 (0.47–1.23)	0.74 (0.59–0.89)	0.75 (0.52–1.00)	0.2614	0.95 (0.64–1.19)	1.03 (0.60–2.15)	0.3479
eGFR (ml/min/1.73 m^2^)	101 (43–145)	91 (74–100)	95 (61–126)	0.0931	91 (71–146)	89 (37–146)	0.6858
CKD stage G1, *n* (%)	20 (71.4)	5 (50.0)	13 (54.2)	0.3224	5 (50.0)	11 (47.8)	0.9999
CKD stage G2, *n* (%)	7 (25.0)	5 (50.0)	11 (45.8)	0.1965	5 (50.0)	7 (30.4)	0.4334
CKD stage G3, *n* (%)	1 (3.6)	0 (0.0)	0 (0.0)	0.5395	0 (0.0)	5 (21.7)	0.2911
ACR (mg/g)	32 (0–201)	43 (23–147)	97 (0–2,436)	0.1732	80 (6–3,083)	235 (0–3,747)	0.5586
Albuminuria, *n* (%)	10 (58.8)	6 (75.0)	12 (66.7)	0.7191	5 (71.4)	14 (77.8)	0.9999
Dialysis/KTx, *n* (%)	0 (0.0)	0 (0.0)	0 (0.0)	0.9999	0 (0.0)	0 (0.0)	0.9999
Lyso-Gb_3_ (ng/ml)	1.9 (0.2–15.8)	1.6 (0.5–9.2)	9.5 (0.4–15.2)	**0**.**0441**	5.2 (0.8–16.0)	69.1 (0.4–197.0)	**0**.**0314**
Lyso-Gb_3_ >reference, *n* (%)	23 (76.7)	6 (66.7)	9 (81.8)	0.7257	8 (88.9)	10 (76.9)	0.6161
IVS (mm)	10.0 (7.0–18.0)	12.5 (9.0–17.0)	15.0 (7.0–22.2)^c^,**	**0**.**0030**	15.0 (11.0–27.0)	14.0 (10.0–30.0)	0.8768
LVH, *n* (%)	8 (29.6)	4 (40.0)	9 (56.3)	0.2932	4 (57.1)	17 (68.0)	0.6675
LVEF (%)	61 (41–81)	63 (52–75)	60 (34–67)	0.6552	54 (34–76)	56 (44–71)	0.6136
LVMi (g/m^2^)	81 (47–155)	88 (49–151)	120 (85–298)^c^,**	**0**.**0173**	138 (76–220)	127 (80–396)	0.9618
NYHA class 1, *n* (%)	27 (90.0)	9 (90.0)	17 (73.9)	0.8824	8 (80.0)	19 (79.2)	0.9999
NYHA class 2, *n* (%)	3 (10.0)	1 (10.0)	5 (21.7)	0.5482	2 (20.0)	3 (12.5)	0.6342
NYHA class 3, *n* (%)	0 (0.0)	0 (0.0)	1 (4.3)	0.4289	0 (0.0)	2 (8.3)	0.9999
SBP (mmHg)	120 (95–160)	132 (105–165)	130 (100–160)	0.3017	130 (110–140)	120 (100–150)	0.6262
DBP (mmHg)	80 (60–102)	80 (65–95)	80 (65–95)	0.9174	80 (70–100)	80 (70–95)	0.5750
ICD/pacemaker, *n* (%)	1 (3.3)	1 (10.0)	1 (4.2)	0.7322	1 (10.0)	0 (0.0)	0.2941
RAAS blocker, *n* (%)	5 (16.1)	5 (50.0)	7 (38.9)	0.0636	7 (70.0)	13 (65.0)	0.9999
Diuretics, *n* (%)	2 (6.5)	2 (20.0)	3 (17.6)	0.3655	2 (20.0)	3 (15.0)	0.9999
Analgesics, *n* (%)	4 (12.9)	3 (30.0)	5 (29.4)	0.2920	2 (20.0)	4 (22.2)	0.9999
DS3 total, score	5 (0–19)	22 (2–34)**	11 (8–27)^c^,**	**0**.**0007**	12 (0–32)	15 (0–34)	0.9309
MSSI total, score	5 (0–27)	17 (6–34)**	13 (3–24)^c^,***	**0**.**0001**	11 (2–34)	12 (2–30)	0.7798
Non-sense mutations, *n* (%)	11 (35.5)	0 (0.0)	15 (62.5)	**0**.**0022**	0 (0.0)	11 (45.8)	**0**.**0135**
p.N215S, *n* (%)	3 (9.7)	1 (10.0)	0 (0.0)	0.2870	7 (70.0)	1 (4.2)	**0**.**0002**
GVUS (p.R118C, p.S126G, p.A143T), *n* (%)	2 (6.5)	5 (50.0)	1 (4.2)	**0**.**0004**	0 (0.0)	3 (12.5)	0.5388

ACR, albumin/creatinine ratio; CKD, chronic kidney disease; DBP, diastolic blood pressure; DS3, disease severity scoring system; eGFR, estimated glomerular filtration rate; ERT, enzyme replacement therapy; FD, Fabry disease; GVUS, genetic variant of unknown significance; ICD, implantable cardioverter device; IVS, interventricular septum thickness; Ktx, kidney transplantation; LVH, left ventricular hypertrophy, defined as LVMi >reference (males: >115 g/m^2^ and females: >95 g/m^2^); LVEF, left ventricular ejection fraction; LVMi, left ventricular mass index; lyso-Gb_3_, globotriaosylsphingosine with an upper limit of normal of 1.8 ng/ml; MSSI, Mainz severity score index; RAAS, renin–angiotensin–aldosterone system; SBP, systolic blood pressure.

The bold values are indicate significant *p*-values, specifically *p* < 0.05.

^a^ Untreated vs. migalastat-treated vs. ERT-treated by the Kruskal–Wallis test.

^b^ Migalastat-treated vs. ERT-treated by the Mann–Whitney test.

^c^ Versus untreated.

* *p* < 0.05.

** *p* < 0.01.

*** *p* < 0.001.

### 3.2. Further baseline cardiac parameters of the study population

**Figure 1:**
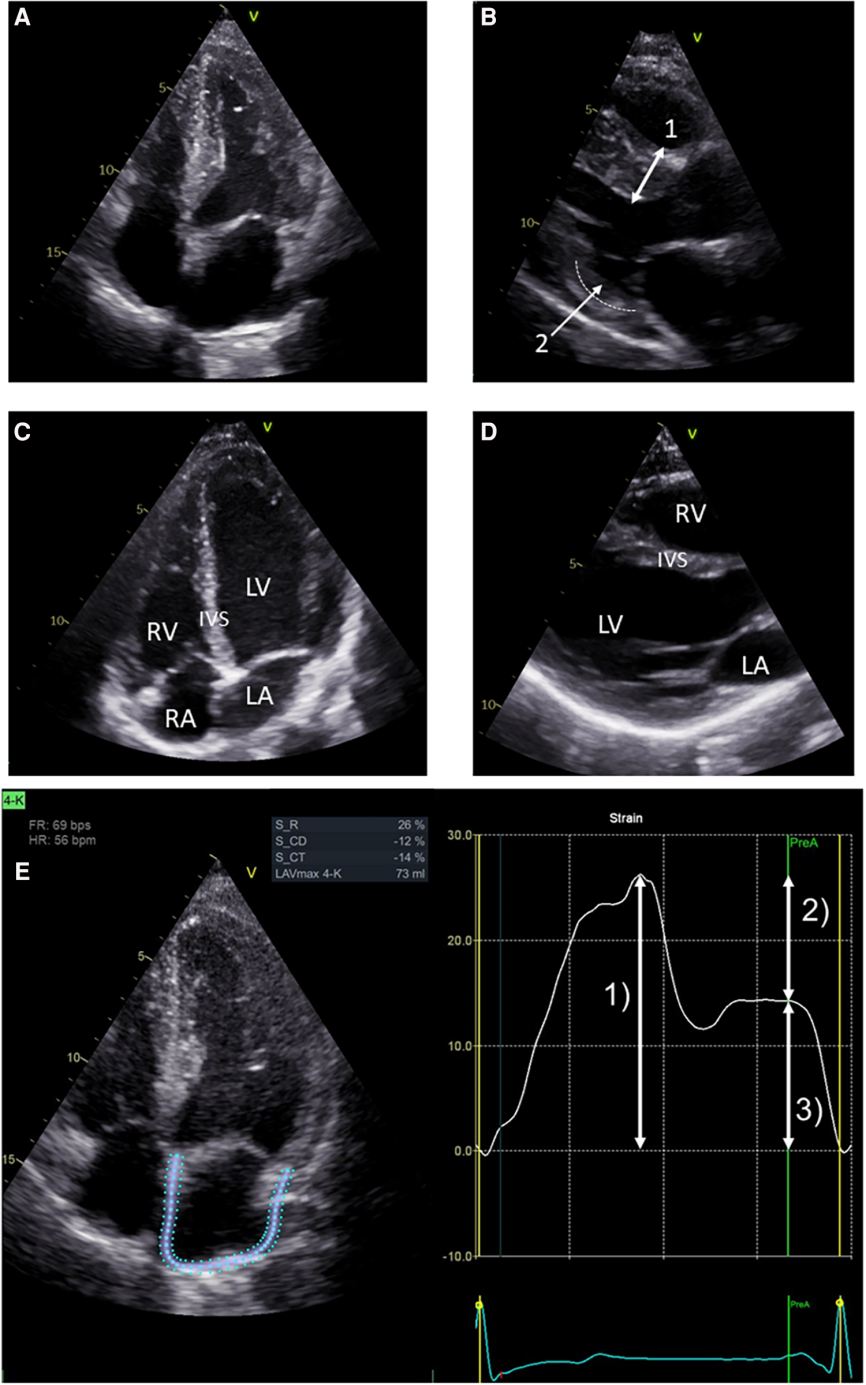
Representative echocardiographic four-chamber views. (**A**) Echocardiographic four-chamber view of a 48-year-old male treated with ERT. Note the LVH of the left ventricle (LV). (**B**) Echocardiographic PLAX view of the same patient. Bidirectional arrow (1) marks septal hypertrophy (measured 15 mm). Arrow (2) marks thinning of the basal infero-lateral wall (dotted line), which is sometimes observed in advanced LVH. (**C**) Echocardiographic four-chamber view of a 26-year-old female from the untreated control group. Left ventricle (LV), right ventricle (RV), left atrium (LA), and right atrium (RA). (**D**) Echocardiographic PLAX view of the same female. This view and (**C**) show an interventricular septum (IVS) of normal size. (**E**) LA strain in echocardiographic four-chamber view on the left side. Note the blue and dotted defined regions of interest (ROI) overlapping the LA walls. Strain values determined by speckle tracking over time are shown on the right. The trigger used for the LA phases is R-R interval. Arrow (1) represents the reservoir function (LaSr) of the LA. Arrow (2) represents the conduction function (LaScd) of the LA. Arrow (3) represents the contraction function (LaSct) of the LA. In the LA cycle, an increase in strain is observed in systole, representing the expansion of the LA. Peak strain is reached at (1). The mitral valve opens, and in diastole, the LA releases blood into the LV represented by the conduit phase. The conduit phase ends by contraction, indicated by the A wave on the ECG. Contraction of the LA enhances LV filling and is represented by (3), which completes the cycle leading back to (1) (analyses for this image used EchoPAC™, version 204, GE).

**Figure 2:**
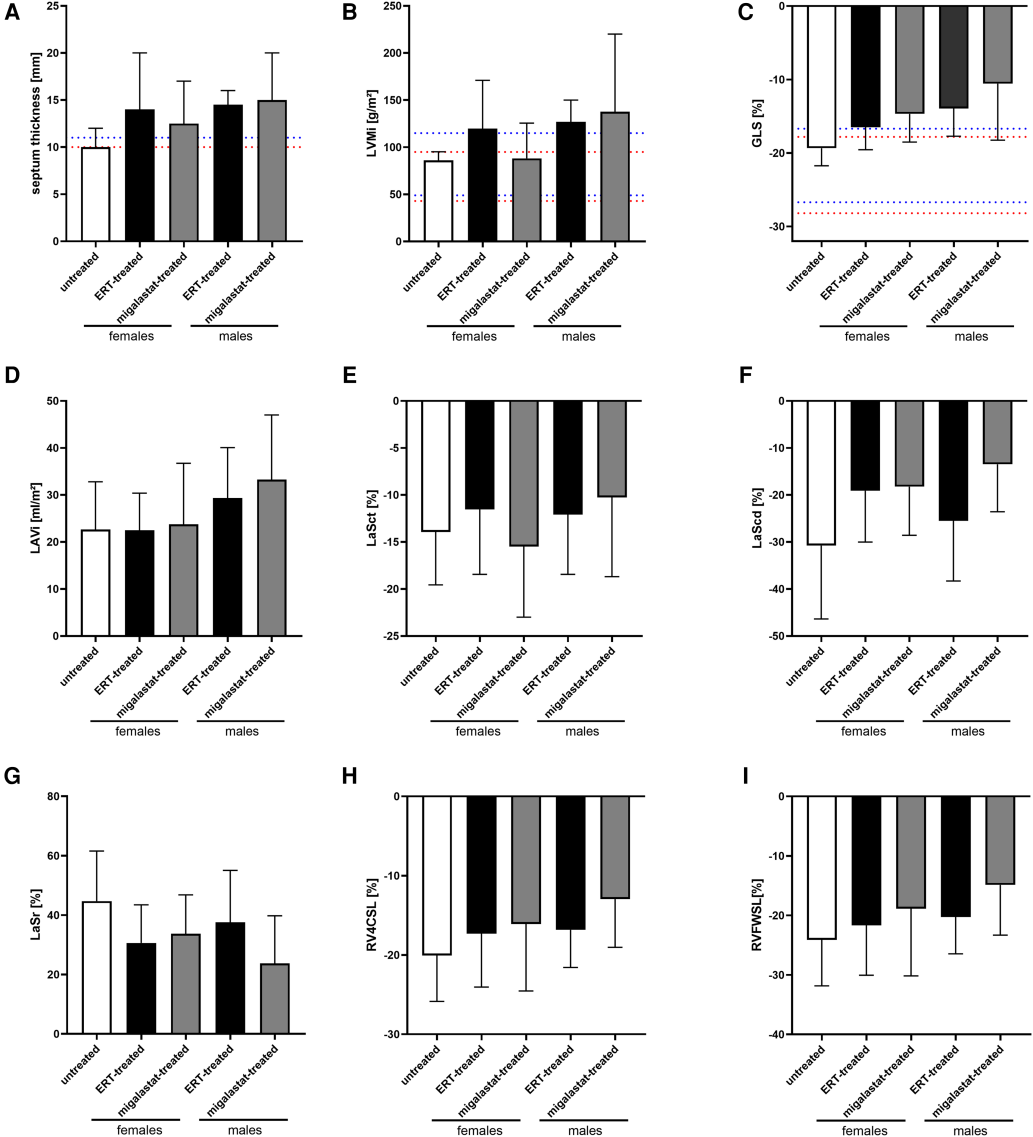
Cardiac parameters in untreated females and ERT- and migalastat-treated females and males at baseline. (**A**) Interventricular septum thickness. (**B**) Left ventricular mass index (LVMi), (**C**) global longitudinal strain average (GLS), (**D**) left atrial (LA) volume index (LAVi), (**E**) LaSct: left atrial contraction “booster” strain, (**F**) LaScd: left atrial conduit strain, (**G**) LaSr: left atrial reservoir strain, (**H**) RV4CSL: right ventricular four-chamber strain, (**I**) RVFWSL: right ventricular free wall longitudinal strain. (**A**) Cut-offs of IVS for LVH are highlighted in red (females, 10 mm) and blue (male, 11 mm). (**B,C**) Normal values (95% CI) according to Lang et al. (20) and Sugimoto et al. [*Eur Heart J Cardiovasc Imaging*. (2017) **18**:833–40] are highlighted in red (females) and blue (males). Data are presented as median ± 95% CI.

Table 2 presents the baseline cardiac parameters and strain analyses. Supplementary Table S2 presents the differences between males and females. Figure 1 shows representative echocardiographic images of LVH in a male patient with FD cardiomyopathy compared with normal cardiac findings in a female patient from the untreated control group. Figure 1E illustrates an example of LAS and the resulting strain measurements drawn as a curve. Intraventricular septum thickness and LVMi values were in the reference range in untreated females (Figures 2A,B). The mean interventricular septum thickness was increased in ERT- and migalastat-treated males and females. The mean LVMi was within the reference range only in migalastat-treated females (Figures 2A,B). Furthermore, the global longitudinal strain (GLS) average values differed significantly between treated and untreated females (Table 2). A comparison to reference values (18) demonstrated that the mean GLS values for untreated females were within the reference range, whereas those in treated patients (females and males) were not (Figure 2C). LAS consists of three phases of the LA: left atrial reservoir function (LaSr), left atrial conduit function (LaScd), and left atrial contraction “booster” strain (LaSct) (Figure 1E). At baseline, LaSr was reduced in the female ERT group compared with the untreated females. A trend toward reduced LaSr was observed in the female migalastat group at baseline. In addition, LaScd was reduced at baseline in FD-specific treated females in comparison to untreated females. Overall, treated males showed lower values for LAS at baseline compared with females. The LA volume index and strain analyses for RV showed no differences between the groups (Figures 2D,H,I). Independent of sex, further cardiac parameters were comparable between migalastat- and ERT-treated patients.

**Table 2:** Baseline values of cardiac parameters.

Group	Females				Males		
	Untreated (*n* = 30)	Migalastat-treated (*n* = 10)	ERT-treated (*n* = 24)	*p*-value^a^	Migalastat-treated (*n* = 10)	ERT-treated (*n* = 24)	*p*-value^b^
LVM (g)	141.4 (78.4 to 296.6)	164.6 (99.7 to 266.9)	196.1 (109.7 to 472.7)*	**0**.**0197**	283 (158 to 444)	272.3 (155.4 to 817.7)	0.6194
LVMi (g/m^2^)	82.9 (46.6 to 155.2)	88.2 (48.6 to 151.3)	114.4 (64.9 to 297.8)*	**0**.**0454**	138 (76 to 220)	130.2 (80.1 to 396.0)	0.8717
LVEDd (mm)	42.0 (34.0 to 60.0)	41.0 (31.0 to 47.0)	40.5 (36.0 to 51.0)	0.5509	49.0 (37.0 to 53.0)	47.0 (30.0 to 56.0)	0.9284
LVESd (mm)	29.0 (34.0 to 38.0)	27.0 (20.0 to 35.0)	30.0 (17.0 to 38.0)	0.4235	30.0 (19.0 to 39.0)	32.5 (20.0 to 46.0)	0.2665
IVS (mm)	10 (7 to 18)	12.5 (9.0 to 17.0)	14.0 (7.0 to 22.0)*	**0**.**0092**	15.0 (11.0 to 27.0)	14.5 (10.0 to 30.0)	0.9131
PW (mm)	9 (5 to 14)	11.5 (7.0 to 15.0)	12.5 (8.0 to 20.0)*	**0**.**0017**	13.0 (7.0 to 25.0)	13.0 (8.0 to 25.0)	0.9920
RVDd (mm)	27.0 (21 to 35)	27.0 (24.0 to 31.0)	28.0 (20.0 to 36.0)	0.9079	28.0 (19.0 to 39.0)	30.0 (19.0 to 36.0)	0.4742
LVEF (%)	61.2 (41.3 to 81.0)	63.0 (51.6 to 74.6)	60.0 (50.0 to 67.0)	0.8017	54.4 (33.9 to 75.6)	62.8 (33.7 to 71.0)	0.7582
LA diameter (mm)	31 (24 to 50)	33 (24 to 40)	35.2 (24.0 to 45.0)	0.2366	38 (25 to 48)	34.0 (24.0 to 55.0)	0.2053
LA volume (ml)	34 (13 to 86)	33 (28 to 93)	46 (18 to 77)	0.6273	52 (42 to 120)	50 (30 to 113)	0.2475
LA volume index (ml/m^2^)	21 (8 to 54)	19 (14 to 55)	23 (11 to 36)	0.9596	25 (20 to 57)	27 (14 to 58)	0.6071
E wave (m/s)	0.74 (0.43 to 1.23)	0.63 (0.46 to 0.98)	0.74 (0.46 to 0.97)	0.2829	0.62 (0.44 to 1.40)	0.75 (0.45 to 1.10)	0.4041
A wave (m/s)	0.49 (0.33 to 0.74)	0.74 (0.40 to 01.00)	0.62 (0.31 to 0.81)	**0**.**0339**	0.48 (0.34 to 5.0)	0.57 (0.27 to 0.90)	0.4692
E/A	1.67 (0.65 to 2.86)	0.78 (0.68 to 1.57)^c^	1.30 (0.65 to 2.10)	**0**.**0050**	1.17 (0.12 to 2.56)	1.35 (0.67 to 2.82)	0.5109
Deceleration velocity (ms)	160 (79 to 265)	144 (115 to 265)^c^	199 (115 to 290)*	**0**.**0144**	155 (72 to 240)	212 (108 to 362)	0.0244
*E*′-lateral	0.13 (0.05 to 0.23)	0.07 (0.06 to 0.10)	0.11 (0.05 to 0.20)	0.1206	0.07 (0.05 to 0.13)	0.12 (0.05 to 0.24)	0.0682
*E*/*E*′-lateral	5.74 (0.296 to 15.4)	8.7 (4.6 to 10.7)	6.7 (3.9 to 13.5)	0.4750	7.2 (4.6 to 23.3)	5.5 (2.8 to 15.6)	0.1164
LaSct (%)	−14.3 (−25.2 to −1.6)	−15.8 (−24.2 to 1.2)	−11.1 (−27.7 to −2.4)	0.2363	−7.8 (−22.4 to 0.57)	−10.0 (−24.0 to −4.6)	0.5101
LaScd (%)	−32.3 (−66.6 to −6.71)	−19.2 (−32.8 to −2.72)	−20.4 (−45.3 to −2.4)	**0**.**0347**	−8.5 (−28.0 to −3.8)	−25.5 (−41.8 to −6.1)	**0**.**0409**
LaSr (%)	43.5 (8.3 to 76.3)	40.7 (12.1 to 50.0)	32.0 (7.6 to 53.3)*	**0**.**0277**	20.1 (7.0 to 47.2)	35.6 (12.9 to 59.5)	0.1383
GLS (%)	−19.3 (−26.4 to −9.34)	−14.7 (−19.1 to −8.3)^c^	−16.7 (−19.6 to −5.3)	**0**.**0086**	−10.6 (18.8 to −5.7)	−13.9 (−23.0 to −6.5)	0.2459
RVFWSL (%)	−25.7 (−36.8 to −6.4)	−18.7 (−41.2 to −0.4)	−23.9 (−30.7 to −3.1)	0.2821	−16.2 (−29.9 to −7.0)	−19.0 (−27.8 to −8.1)	0.3786
RV lateral diameter (mm)	5 (3 to 7)	5 (4 to 7)	5 (3 to 8)	0.6598	7 (4 to 9)	6 (4 to 11)	0.5467
NT-proBNP (pg/ml)	54 (30 to 659)	196 (50 to 937)	95 (38 to 3,712)	0.0716	295 (38 to 2,203)	79 (30 to 8,193)	0.3942

BSA, body surface area; LVM, left ventricular mass; LVMi, left ventricular mass index; LVEDd, left ventricular end-diastolic diameter; LVESd, left ventricular end-systolic diameter; IVS, interventricular septum thickness; PW, posterior wall thickness; RVDd, right ventricular diastolic diameter; LVEF, left ventricular ejection fraction; LaSct, left atrial contraction “booster” strain; LaScd, left atrial conduit strain; LaSr, left atrial reservoir strain; GLS, global longitudinal strain average; RVFWSL, right ventricular free wall longitudinal strain.

The bold values are indicate significant *p*-values, specifically *p* < 0.05.

^a^ Untreated vs. migalastat-treated vs. ERT-treated by the Kruskal–Wallis test.

^b^ Migalastat-treated vs. ERT-treated by the Mann–Whitney test.

^c^ Versus ERT-treated.

* *p* < 0.05 tested via one sample *t* or Wilcoxon test.

### 3.3. Outcomes for cardiac parameters

**Figure 3:**
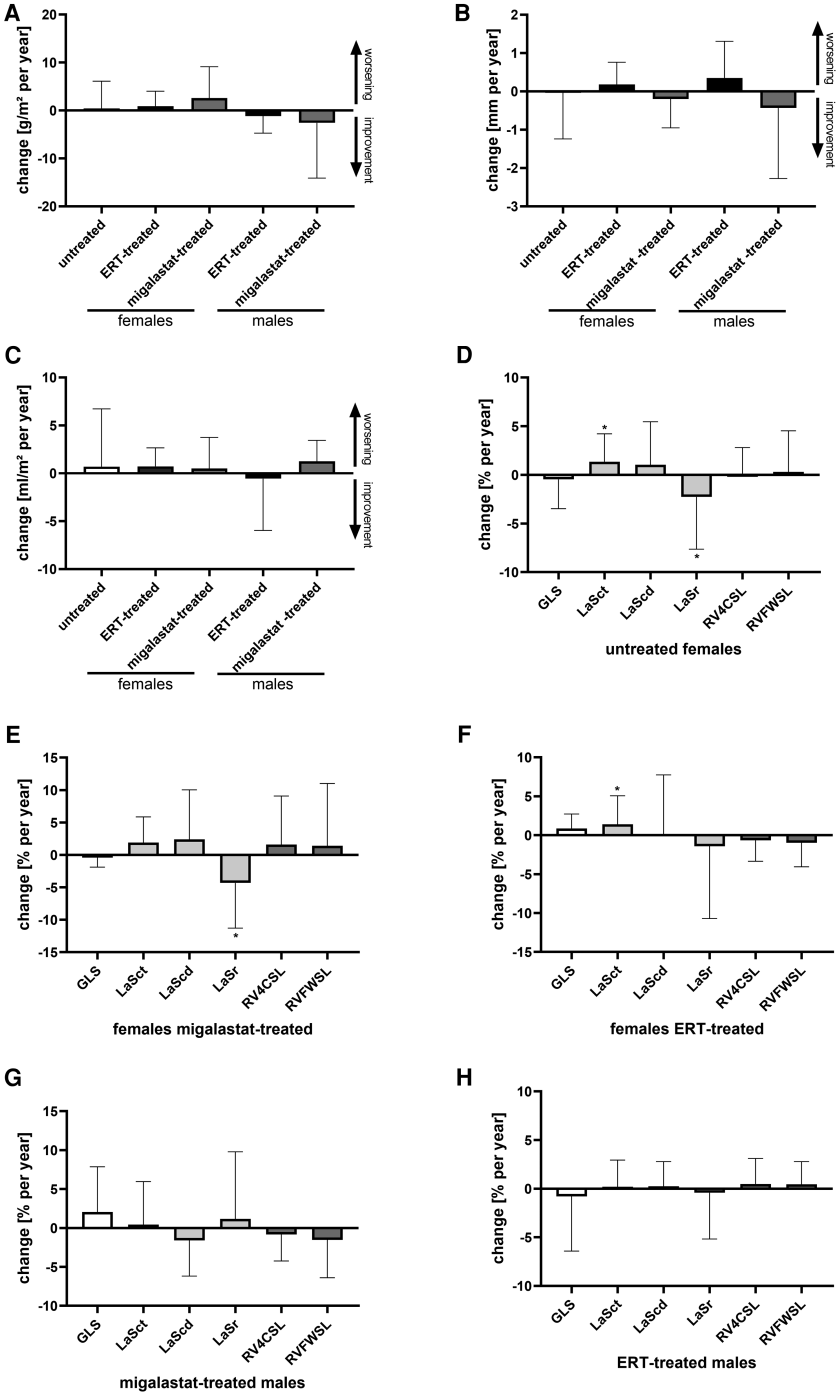
Outcomes for cardiac parameters in untreated females and ERT- and migalastat-treated females and males. (**A**) Yearly change of left ventricular mass index in untreated females and females and males treated with ERT and migalastat. (**B**) Yearly change of interventricular septum thickness in untreated females, and females and males treated with ERT and migalastat. (**C**) Yearly change of left atrial (LA) volume index in untreated females, and females and males treated with enzyme replacement therapy (ERT) and migalastat. (**D**) Yearly changes of global longitudinal strain (GLS) and right ventricle (RV) strains in untreated females. (**E**) Yearly changes of GLS and RV strains in migalastat-treated females. (**F**) Yearly changes of GLS and RV strains in ERT-treated females. (**G**) Yearly changes of GLS and RV strains in migalastat-treated males. (**H**) Yearly changes of GLS and RV strains in ERT-treated males. (**D**–**H**) Negative changes for GLS, LaSct, LaScd, RV4CSL, and RVFWSL mean an improvement. An asterisk marks significant changes. *p < 0.05. LaSct: left atrial contraction “booster” strain; LaScd: left atrial conduit strain; LaSr: left atrial reservoir strain; RV4CSL: right ventricular fourchamber strain; GLS: global longitudinal strain; RVFWSL: right ventricular free wall longitudinal strain; ERT: enzyme replacement therapy; LVH: left ventricular hypertrophy.

Table 3 presents the outcomes for cardiac parameter and strain analyses. Supplementary Table S3 presents the differences between treated males and females. Independent of treatment, yearly changes in cardiac parameters including LVMi in females and males were stable, pointing toward disease stabilization (Table 3, Figure 3). Strain analyses revealed mainly stable values in untreated females. However, the LaSr values showed a significant worsening (*p* = 0.0376). In addition, LaSct showed some deterioration over time (*p* = 0.0280) (Figure 3D) in that group. A worsening in LaSr was also observed in migalastat-treated females (*p* = 0.0371), while all other parameters remained stable (Figure 3E). ERT-treated females were also mainly stable and only slightly deteriorated for LaSct (*p* = 0.0348; Figure 3F). GLS was stable (*p* > 0.05) in untreated females (0.03% per year), migalastat-treated females (0.07% per year), and migalastat-treated males (1.36% per year) (Figures 3D,E,G). GLS was also stable (*p* > 0.05) in ERT-treated females (0.85% per year) and males (0.37% per year) (Figures 3F,H). 2DSTE of the RV free wall showed no difference at baseline and in follow-up.

**Table 3:** Outcomes for cardiac parameters.

Group	Females				Males		
	Untreated (*n* = 30)	Migalastat-treated (*n* = 10)	ERT-treated (*n* = 24)	*p*-value^a^	Migalastat-treated (*n* = 10)	ERT-treated (*n* = 24)	*p*-value^b^
Parameter (change per year)							
LVM (g)	0.52 (−96.6 to 71.5)	5.96 (−62.8 to 16.4)	1.90 (−44.3 to 24.6)	0.8108	−14.00 (−127.8 to 68.8)	0.73 (−36.5 to 26.4)	0.1313
LVMi (g/m^2^)	1.44 (−44.6 to 45.6)	2.57 (−36.8 to 10.0)	0.86 (−10.9 to 13.9)	0.8990	−7.2 (−14.2 to 27.9)	−1.17 (−15.6 to 15.7)	0.4195
LVEDd (mm)	−0.34 (−10.4 to 7.5)	0.47 (−7.6 to 5.1)	0.0 (−3.9 to 3.5)	0.9451	−1.44 (−13.7 to 3.6)*	−0.27 (−4.0 to 2.1)	0.0635
LVESd (mm)	−0.43 (−8.6 to 6.0)	1.03 (−5.6 to 3.3)	0.0 (−1.6 to 9.2)	0.5597	−2.2 (−9.3 to 3.7)	0.03 (−5.5 to 9.9)	0.0787
IVS (mm)	0.0 (−3.0 to 3.0)	−0.29 (−1.2 to 1.6)	0.0 (−0.6 to 2.1)	0.2547	0.00 (−5.1 to 1.0)	0.11 (−1.7 to 3.5)	0.4634
PW (mm)	0.0 (−1.6 to 2.5)	0.0 (−1.1 to 2.4)	0.0 (−2.3 to 2.1)	0.9317	0.00 (−8.6 to 1.6)	0.11 (−1.8 to 1.5)	0.9211
RVDd (mm)	−0.58 (−3.8 to 4.0)*	0.46 (−3.1 to 3.3)	−0.48 (−5.2 to 1.5)	0.2145	1.45 (−9.9 to 7.1)	0.29 (−1.5 to 1.7)	0.1473
LVEF (%)	0.7 (−14.9 to 9.5)	−0.29 (−8.5 to 15.3)	0.1 (−3.5 to 26.8)	0.9749	0.9 (−18.4 to 8.9)	0.77 (−7.6 to 6.2)	0.7804
LA diameter (mm)	0.0 (−9.5 to 10.0)	0.70 (−1.8 to 7.2)	0.0 (−2.1 to 3.5)	0.6490	0.50 (−2.4 to 8.8)	−0.11 (−5.3 to 1.9)	0.2633
LaSct (%)	0.26 (−2.57 to 7.12)*	0.98 (−2.45 to 11.77)	0.85 (−7.19 to 7.95)*	0.8633	0.87 (−11.88 to 7.10)	0.28 (−3.51 to 4.81)	0.9688
LaScd (%)	0.78 (−8.55 to 12.35)	0.87 (−11.1 to 17.6)	0.10 (−6.56 to 25.58)	0.6060	−0.81 (−10.11 to 4.49)	0.18 (−13.91 to 3.93)	0.3669
LaSr (%)	−1.88 (−17.9 to 10.6)*	−1.08 (−20.7 to 2.23)*	−0.96 (−31.95 to 12.68)	0.6703	0.20 (−11.59 to 19.26)	−0.26 (−6.17 to 9.92)	0.7623
LA volume index (ml/m^2^)	0.02 (−11.06 to 18.43)	1.95 (−4.43 to 5.16)	0.89 (−2.58 to 4.56)	0.6869	1.76 (−1.24 to 4.48)	−0.64 (−16.8 to 11.28)	0.0871
E wave (m/s)	0.00 (−0.2 to 0.2)	0.03 (−0.1 to 0.1)^c^	−0.03 (−0.1 to 0.0)*	**0**.**0180**	0.00 (−0.1 to 0.2)	−0.01 (−0.1 to 0.1)	0.4615
A wave (m/s)	0.01 (−0.1 to 0.2)	0.06 (−0.2 to 0.3)	0.0 (−0.1 to 0.1)	0.1175	−0.02 (−1.5 to 0.1)	0.0 (−0.1 to 0.1)	0.5383
E/A	0.03 (−0.6 to 0.1)*	−0.03 (−0.4 to 0.4)	−0.03 (−0.1 to 4.)	0.8327	0.14 (−0.3 to 0.5)	0.0 (−0.4 to 0.4)	0.2259
Deceleration velocity (ms)	7.5 (−43.9 to 36.8)	15.1 (−44.0 to 63.0)	−0.6 (−53.3 to 32.3)	0.5491	0.6 (−36.0 to 68.6)	−0.83 (−40.4 to 61.5)	0.6211
*E*′ lateral	0.0 (−0.1 to 0.1)	0.00 (0.0 to 0.01)	0.0 (0.0 to 0.0)	0.1133	0.01 (−0.02 to 0.02)	0.0 (−0.03 to 0.01)*	0.2205
GLS (%)	0.03 (−14.1 to 2.9)	0.07 (−2.9 to 1.7)	0.85 (−0.9 to 6.6)*	0.1532	1.38 (−6.6 to 13.3)	0.37 (−20.7 to 3.6)	0.2895
RVFWSL (%)	0.4 (−8.8 to 8.6)	−1.5 (−8.6 to 25.7)	−0.3 (−7.3 to 3.0)	0.4413	−1.3 (11.0 to 5.7)	0.4 (−4.3 to 6.3)	0.4820

LVM, left ventricular mass; LVMi, left ventricular mass index; LVEDd, left ventricular end-diastolic diameter (ED); LVESd, left ventricular end-systolic diameter; IVS, interventricular septum thickness; PW, posterior wall thickness; RVDd, right ventricular diameter diastolic; LVEF, left ventricular ejection fraction; LAD, left atrial diameter; RVFWSL, right ventricular free wall longitudinal strain.

The bold values are indicate significant *p*-values, specifically *p* < 0.05.

^a^ Untreated vs. migalastat-treated vs. ERT-treated by Kruskal–Wallis test.

^b^ Migalastat-treated vs. ERT-treated by Mann–Whitney test.

^c^ Versus ERT-treated.

* *p* < 0.05.

** *p* < 0.01.

*** *p* < 0.001.

**Figure 4:**
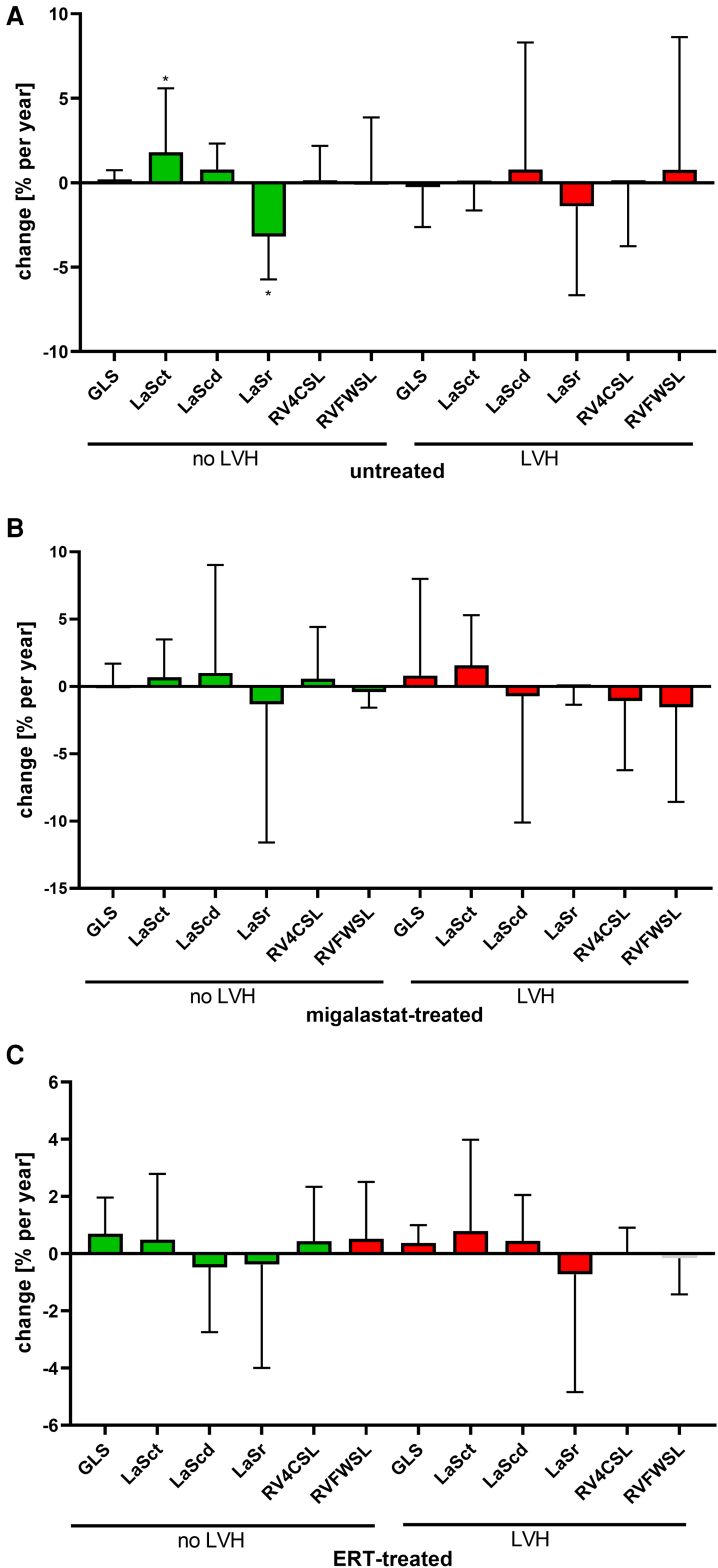
Impact of LVH at baseline on cardiac parameters. (**A**) Changes in untreated patients. (**B**) Changes in migalastat-treated patients. (**C**) Changes in ERT-treated patients. The presence of left ventricular hypertrophy (LVH) was defined as left ventricular mass index >reference (females >95 g/m², males >115 g/m²). (**A**–**C**) Negative changes for GLS, LASct, LAScd, RV4CSL, and RVFWSL mean an improvement. LaSct: left atrial contraction “booster” strain; LaScd: left atrial conduit strain; LaSr: left atrial reservoir strain; RV4CSL: right ventricular four-chamber strain; GLS: global longitudinal strain; RVFWSL: right ventricular free wall longitudinal strain; ERT: enzyme replacement therapy; LVH: left ventricular hypertrophy. An asterisk marks significant changes. *p < 0.05.

Migalastat- and ERT-treated males remained stable over time (Figures 3G,H). The NYHA classifications in all patients were also stable (all *p* > 0.05). To analyze the impact of LVH on strain outcomes, the patients were stratified according to the presence of LVH at baseline (defined as LVMi >reference as described in the methods). Untreated females without LVH showed a significant worsening of LaSr and LaSct (both *p* < 0.05; Figure 4A). Interestingly, untreated females with LVH were mainly stable but only showed a significant increase of LVMi over time (*p* < 0.05; Supplementary Figure S4). Patients receiving migalastat or ERT showed stable values, independent of LVH at baseline (Figures 4B,C; Supplementary Figure S4).

The plasma lyso-Gb_3_ levels in untreated females (−0.3 ng/ml per year) and migalastat-treated females (0.1 ng/ml per year) and males (−0.6 ng/ml per year) remained stable (all *p* > 0.05). Vice versa, ERT-treated females (−1.2 ng/ml per year) and males (−14.2 ng/ml per year) presented with a significant reduction of lyso-Gb_3_ (*p* = 0.0122 and *p* = 0.0171, respectively).

To analyze if echo parameters might correlate with disease markers in treated patients, we performed simple regression analyses with NT-proBNP, albumin–creatinine ratio (ACR), and lyso-Gb_3_ values at baseline (Supplementary Table S4). In ERT-treated patients, only eGFR values were significantly correlated (negative) with NT-proBNP and ACR. In migalastat-treated patients, high NT-proBNP values correlated well with worse LVMi, eGFR, LaSct, LaScd, LaSr, GLS, and RVFWSL values (Supplementary Table S4).

Since treatment effects of the renin–angiotensin–aldosterone system (RAAS) blockers in patients with FD are yet elusive, we analyzed the effect of RAAS treatment on the main cardiovascular parameters assessed (LVMI, GLS, RVFWSL, RV4CSL, LaSct, LaScd, and LaSr) in migalastat- and ERT-treated patients over time (Supplementary Figure S5). Independent of the treatment with migalastat or ERT, we did not observe any effect of RAAS treatment on yearly changes of GLS, RVFWSL, RV4CSL, LaSct, LaScd, and LaSr between RAAS-untreated and RAAS-treated patients (Supplementary Figure S5).

## 4. Discussion

Although cardiac involvement in FD presents a major course of morbidity and mortality, data on recent and promising echocardiographic methods are limited. This study investigates the effects of FD-specific treatment using ERT and chaperone therapy on LA function using 2DSTE. Our main findings were as follows: (1) As expected, patients with FD-specific treatment had increased LVMi and frequency of LVH at baseline, whereas untreated control patients showed normal baseline values. Importantly, LVMi did not increase during follow-up in treated FD patients or untreated controls. (2) GLS was decreased at baseline in patients with FD-specific treatment but stable over time. (3) Patients with FD-specific treatment showed reduced LAS at baseline and stabilization of LAS derived from 2DSTE.

### 4.1. LVMi in FD

In general, an elevated LVMi suggests a poorer prognosis, as it correlates with reduced LV ejection fraction (LVEF) and increased morbidity post–myocardial infarction (25, 26).

Our patients treated with FD-specific therapy showed an increased LVMi at baseline compared with the untreated control group. This might be explained by the time of diagnosis and the resulting delay in initiating FD-specific therapy. LVMi is an established parameter of LVH and for FD progression and outcome (27, 28). During follow-up, we did not observe significant LVMi changes, consistent with data from the Fabry Outcome Survey (FOS) reported by Kampmann et al. for agalsidase-alfa (29). For agalsidase-beta (30), a stabilization of wall thickness was reported in patients with therapy initiation before 40 years of age, but wall thickness increased when therapy was initiated in older patients. Regarding the effects of migalastat therapy, the initial studies (ATTRACT and FACETS) and a German study with a real-world design showed a decrease in LVMi over time (31–33). In our study, there was no decrease in LVMi with migalastat. One reason could be that the LVMi of our treated patients at baseline was higher compared with FAMOUS-24 (FAMOUS-24: mean LVMi in women of 78 vs. 88.2 g/m^2^ and in men of 117 vs. 138 g/m^2^). A comparison of LVMi in our study to ATTRACT and FACETS is not easily transferable because sex-specific measurements were not obtainable [ATTRACT mean LVMi for both sexes: 97.5 g/m^2^; FACETS mean LVMi in the migalastat group 93.3 ± 30 (SD) and placebo–migalastat group 101.7 ± 37 (SD)].

### 4.2. GLS in FD

Using GLS in heart failure and FD particularly has been demonstrated for diagnosing and monitoring the disease progression. In chronic heart failure, GLS has demonstrated a prognostic value for worsening heart failure (34). Moreover, GLS predicts heart failure in coronary artery disease patients and adverse events in non-ischemic cardiomyopathies (35, 36). There is evidence that reduced GLS is an early marker of FD cardiomyopathy (37) and also predicts outcomes (38). We show that patients treated with either ERT or chaperone therapy have reduced GLS, with male patients having the most reduced (worst) GLS at baseline in both treatment groups. Over time, GLS did not worsen in the control or treatment groups. Thus, we conclude that GLS was stable. Segmental strain patterns of the LV may indicate FD diagnosis (12). However, our treated patients did not show a significant regional strain reduction pattern in the basal posterolateral segments at baseline.

### 4.3. LAS in FD

LAS is influenced by structural properties, filling pressure, and diastolic function of the LV and LA (39) and consists of three components, namely, LaSr, LaScd, and LaSct. All three baseline LAS values of the treatment groups were reduced (worse) compared with those of the control group and healthy subjects from the literature (40). With regard to the time course of the ERT-treated group, LAS values were stable and did not worsen over time. In migalastat-treated females, LaSr worsened, while other strain values remained stable. However, in migalastat-treated males, all strain values remained stable. The control group also showed a worsening in LaSr.

Of note, LAS measured in our control group was in the reported normal ranges in a meta-analysis by Pathan et al. and in a community-based longitudinal cohort study from Copenhagen (41), emphasizing its function as a control group for LAS.

In FD, Pichette et al. demonstrated reduced LAS at baseline in all three phases, including LaSr, LaScd, and LaSct, in patients receiving ERT and a natural history cohort (42). In comparison to our observation, Pichette et al. described improvements in LaSr and in some cases LaScd and LaSct after 12 months of ERT. Compared with the study by Pichette et al., our ERT group showed a higher LVMi at baseline, which could be explained by a more fixed cardiac structural change. The worsening in LaSr in migalastat-treated females could be attributed to a higher age median. The observed reduction of LaSr in our untreated group cannot be fully explained, but possible contributing factors may include aging (41), comorbidities such as arterial hypertension, or a subclinical course of FD. However, this assumption should be taken with caution and requires further verification.

LAS is emerging as a prognosticator for the recurrence of atrial fibrillation and cardiovascular morbidity (43–45). Similar to GLS, LAS has shown value in diagnosing FD and allowing differentiation to other cardiomyopathies with a hypertrophic phenotype (46). Because FD is a rare disease that shows a hypertrophic phenotype in its course, it seems possible to consider observations from other cardiomyopathies with a hypertrophic phenotype (such as HCM and amyloidosis) as similar (47). Recently, LAS impairment and correlation with FD severity have also been described as measured using cardio-MRI (48), highlighting the usefulness of LAS, even when acquired by another modality.

### 4.4. Biomarkers

We analyzed correlations between proteinuria, NT-proBNP, and lyso-Gb_3_ with echocardiographic measures, as detailed in Supplementary Table S4. The NT-proBNP values correlated with all strain values and LVMI in migalastat-treated patients (both genders combined because of limited sample sizes); this was not seen in ERT patients. The migalastat group, being older, could influence this correlation (49). While NT-proBNP is crucial for diagnosing heart failure, in general, its role in tracking disease progression does not offer a clear threshold (50). Therefore, we view this correlation as hypothesis-generating.

In addition, ACR correlated with LVMi in the migalastat group, possibly because of age and arterial hypertension. In the same group, lyso-Gb_3_ correlated with LVMi and GLS, although defining a harmful lyso-Gb_3_ threshold remains challenging.

### 4.5. Age

The life expectancy of patients with FD has increased over the years because of FD-specific treatments, advances in concomitant medications, and better screening protocols that identify patients at risk much earlier. In our cohort, the highest proportion of patients with an age over 50 years was found in the migalastat group (65%). This proportion was much lower in the ERT-treated patients and the untreated group (33.3% and 26.7%, respectively). Due to the scope of our single-center study, our analysis of age effects was limited. Future studies focusing on therapy effects especially within the elderly are required. In this respect, large registries such as the FOS, Fabry Registry, and followME registry would suit this purpose.

### 4.6. Heart failure medication

In our study, current cardiological guidelines guided the prescription of RAAS blockers (51, 52), recommending them as a class IIb indication for patients with LVEF greater than 40%. Furthermore, RAAS blockers are indicated for LVEF less than 50%, which is an approximation of the guidelines for HCM (53). Because most patients had LVEF greater than 50%, not all were taking RAAS blockers. In addition, some patients were excluded because of intolerance to RAAS blockers, such as hypotension. Nephrological indications, such as renal insufficiency, were also determinants for prescribing these drugs. In accordance with the class IIb indication (52), we did not observe the effects of RAAS blockers on the assessed cardiovascular parameters in our cohort.

### 4.7. FD cardiomyopathy and effects of treatment

The accumulation of Gb_3_ in cardiomyocytes is observed early in the life of FD patients (54), but the extent of FD cardiomyopathy and fibrosis is unlikely to be explained by Gb_3_ accumulation alone (11). Gb_3_ accumulation affects many heart structures, including the LA at the histologic level (55). Pathological lysosomal storage of Gb_3_ in cardiomyocytes triggers a cascade leading to extracellular remodeling of the heart in FD patients because of inflammation and accumulation of fibrosis (56, 57). Increasing and severe LVH because of fibrosis is also observed in other cardiomyopathies with a hypertrophic phenotype, such as amyloidosis, aortic valve stenosis, and HCM. FD cardiomyopathy progresses over time (58). Once cardiac fibrosis has occurred, reversal seems to be rarely possible because of the limited regenerative capacity of the heart (59). This may explain why we did not observe a reversal of LVMi and GLS and recovery of LAS.

Although we observed the patients for up to 81 months, this time frame was likely insufficient to detect hard endpoints (such as death and cardiac infarction), given their low probability of occurrence. Since FD shows a relatively slow disease progression (compared with other lysosomal storage diseases), longer observational periods are required to analyze if the echo parameters are potential predictors for hard endpoints in FD.

Initiating treatment with ERT or migalastat halts or significantly slows cardiac progression in the form of LVH, development of diastolic dysfunction, and deterioration of LA function. Our study highlights that FD-specific therapies can stop the progression of FD cardiomyopathy, which is important because FD cardiomyopathy is considered a major cause of death in FD patients. The early initiation of FD-specific therapies is important because the existing changes in FD cardiomyopathy are predominantly irreversible.

### 4.8. Limitations

A strength of our study is the large cohort of 68 patients available for a long follow-up of up to 81 months. A limitation of our study is that image acquisition was performed on different echomachines (GE and Philips). However, post-processing and analysis were performed using the same software (TOMTEC), allowing strain value comparison. The relatively low patient number because of the five different groups might be a limitation by reducing the statistical power. Therefore, our data should be interpreted carefully, and additional studies with larger patient cohorts are required to strengthen our preliminary results.

Our control group consists of females who were younger than the treated groups. These young females may not have been treated because FD has not yet manifested. In addition, with age, diastolic function may deteriorate because of age-related symptoms or common comorbidities such as arterial hypertension. Due to present mutations of different pathogenetic severity, FD patients exhibit various disease courses. Furthermore, since the start of observation, some genetic variants have been reclassified over time. In the migalastat group, there is a clustering of the p.N215S mutation in males. To some extent, all these points are because of the real-world design of our study and the fact that FD is a rare disease. We are aware that some of the included patients had GVUS, for which therapy is currently questionable (60), which might be a limitation.

As a multisystemic disease, FD also manifests in other organs such as the kidney. An example is chronic kidney disease because FD may lead to arterial hypertension and volume overload, eventually resulting in impaired LAS (61). However, the number of patients treated for arterial hypertension was evenly distributed in our groups. We did not observe any severe and untreated arterial hypertension in our patients.

## 5. Conclusion

In patients with FD treated with either ERT or chaperone therapy, LAS measured by 2DSTE are stable over time. LAS might also be an appropriate follow-up disease marker as in other cardiac diseases. Further investigation is needed to assess whether LAS is useful in deciding whether to initiate FD therapy.

## Data Availability

The original contributions presented in the study are included in the article/**Supplementary Material**, further inquiries can be directed to the corresponding author.
