# Supplementary material for: Impact of enzyme replacement therapy and migalastat on left atrial strain and cardiomyopathy in patients with Fabry disease
Source: Front Cardiovasc Med. 2023 Nov 2;10:1223635. doi: 10.3389/fcvm.2023.1223635 (PMC10656767; doi:10.3389/fcvm.2023.1223635)
Supplement: Supplementary file 1 [file Table1.docx]

Supplementary Material

Table of contents

[1 Supplementary tables 2](#_Toc144817535)

[1.1 Supplemental Table 1: Comparison of baseline characteristics of the study cohort between females and males. 2](#_Toc144817536)

[1.2 Supplemental Table 2: Comparison of baseline values of cardiac parameters between females and males. 4](#_Toc144817537)

[1.3 Supplemental Table 3: Comparison of outcomes for cardiac parameters between females and males. 5](#_Toc144817538)

[1.4 Supplemental table 4: Correlation between NT-proBNP, ACR and lyso-Gb_3_ with cardiac parameters at baseline. 6](#_Toc144817539)

[2 Supplementary Figures 7](#_Toc144817540)

[2.1 Supplemental Figure 1: Overview of the study population and their analyses. 7](#_Toc144817541)

[2.2 Supplemental Figure 2: Intraobserver variability 8](#_Toc144817542)

[2.3 Supplemental Figure 3: Interobserver variability 9](#_Toc144817543)

[2.4 Supplemental Figure 4: Impact of LVH at baseline on left ventricular mass index. 10](#_Toc144817544)

[2.5 Supplemental Figure 5: Effects of RAAS-blockers on cardiovascular parameters 11](#_Toc144817545)

# Supplementary tables

| Supplemental Table 1: Comparison of baseline characteristics of the study cohort between females and males. | | | | | | |
| --- | --- | --- | --- | --- | --- | --- |
| **group** | **migalastat-treated** | | | **ERT-treated** | | |
|  | **females [n=10]** | **males [n=10]** | **p-value** | **females [n=24]** | **males [n=24]** | **p-value** |
| **age, years** | 62 [44-72] | 52 [16-68] | 0.2161 | 48 [25-71] | 36 [18-65] | 0.0637 |
| **mean follow-up, months** | 28±10 | 26±6 | 0.5900 | 75±40 | 81±39 | 0.6317 |
| **pre-treated with ERT, n (%)** | 4 (40.0) | 4 (40.0) | 0.9999 | 1 (4.2) | 0 (0.0) | 0.9999 |
| **angioceratoma, n (%)** | 2 (20.0) | 1 (10.0) | 0.9999 | 9 (39.1) | 15 (65.2) | 0.1392 |
| **edema, n (%)** | 1 (10.0) | 2 (20.0) | 0.9999 | 0 (0.0) | 5 (20.8) | **0.0496** |
| **diarrhea, n (%)** | 4 (44.4) | 0 (0.0) | **0.0325** | 6 (25.0) | 8 (36.4) | 0.5252 |
| **abdominal pain, n (%)** | 5 (50) | 4 (40.0) | 0.9999 | 6 (25.0) | 7 (30.4) | 0.7516 |
| **FD-specific pain, n (%)** | 6 (60.0) | 6 (60.0) | 0.9999 | 16 (66.7) | 14 (58.3) | 0.7661 |
| **hypohidrosis, n (%)** | 2 (20.0) | 2 (20.0) | 0.9999 | 8 (34.8) | 13 (54.2) | 0.2443 |
| **creatinine, mg/dl** | 0.74 [0.59-0.89] | 0.95 [0.64-1.19] | **0.0137** | 0.75 [0.52-1.00] | 1.03 [0.60-2.15] | **0.0003** |
| **eGFR, ml/min/1.73 m²** | 91 [74-100] | 91 [71-146] | 0.7959 | 95 [61-126] | 89 [37-146] | 0.7557 |
| **CKD stage G1, n (%)** | 5 (50.0) | 5 (50.0) | 0.9999 | 13 (54.2) | 11 (47.8) | 0.7732 |
| **CKD stage G2, n (%)** | 5 (50.0) | 5 (50.0) | 0.9999 | 11 (45.8) | 7 (30.4) | 0.3715 |
| **CKD stage G3, n (%)** | 0 (0.0) | 0 (0.0) | 0.9999 | 0 (0.0) | 5 (21.7) | **0.0219** |
| **ACR, mg/g** | 43 [23-147] | 80 [6-3083] | 0.7209 | 97 [0-2436] | 235 [0-3747] | 0.1882 |
| **albuminuria, n (%)** | 6 (75.0) | 5 (71.4) | 0.9999 | 12 (66.7) | 14 (77.8) | 0.7112 |
| **dialysis/KTx, n (%)** | 0 (0.0) | 0 (0.0) | 0.9999 | 0 (0.0) | 0 (0.0) | 0.9999 |
| **lyso-Gb_3_, ng/ml** | 1.6 [0.5-9.2] | 5.2 [0.8-16.0] | **0.0364** | 9.5 [0.4-15.2] | 69.1 [0.4-197.0] | **0.0109** |
| **lyso-Gb_3_ > reference, n (%)** | 6 (66.7) | 8 (88.9) | 0.5765 | 9 (81.8) | 10 (76.9) | 0.9999 |
| **IVS, mm** | 12.5 [9.0-17.0] | 15.0 [11.0-27.0] | 0.2990 | 15.0 [7.0-22.2] | 14.0 [10.0-30.0] | 0.8866 |
| **LVH, n (%)** | 4 (40.0) | 4 (57.1) | 0.6372 | 9 (56.3) | 17 (68.0) | 0.5170 |
| **LVEF, %** | 63 [52-75] | 54 [34-76] | 0.2224 | 60 [34-67] | 56 [44-71] | 0.3169 |
| **LVMi, g/m²** | 88 [49-151] | 138 [76-220] | 0.1088 | 120 [85-298] | 127 [80-396] | 0.4983 |
| **NYHA class 1, n (%)** | 9 (90.0) | 8 (80.0) | 0.9999 | 17 (73.9) | 19 (79.2) | 0.9999 |
| **NYHA class 2, n (%)** | 1 (10.0) | 2 (20.0) | 0.9999 | 5 (21.7) | 3 (12.5) | 0.7049 |
| **NYHA class 3, n (%)** | 0 (0.0) | 0 (0.0) | 0.9999 | 1 (4.3) | 2 (8.3) | 0.9999 |
| **SBP, mmHg** | 132 [105-165] | 130 [110-140] | 0.3850 | 130 [100-160] | 120 [100-150] | 0.2589 |
| **DBP, mmHg** | 80 [65-95] | 80 [70-100] | 0.5309 | 80 [65-95] | 80 [70-95] | 0.6513 |
| **ICD/pacemaker, n (%)** | 1 (10.0) | 1 (10.0) | 0.9999 | 1 (4.2) | 0 (0.0) | 0.999 |
| **RAAS-blocker, n (%)** | 5 (50.0) | 7 (70.0) | 0.6499 | 7 (38.9) | 13 (65.0) | 0.1927 |
| **diuretics, n (%)** | 2 (20.0) | 2 (20.0) | 0.9999 | 3 (17.6) | 3 (15.0) | 0.9999 |
| **analgesics, n (%)** | 3 (30.0) | 2 (20.0) | 0.9999 | 5 (29.4) | 4 (22.2) | 0.7112 |
| **DS3 total, score** | 22 [2-34] | 12 [0-32] | 0.5898 | 11 [8-27] | 15 [0-34] | 0.2393 |
| **MSSI total, score** | 17 [6-34] | 11 [2-34] | 0.7941 | 13 [3-24] | 12 [2-30] | 0.9089 |
| **nonsense mutations, n (%)** | 0 (0.0) | 0 (0.0) | 0.9999 | 15 (62.5) | 11 (45.8) | 0.3852 |
| **p.N215S, n (%)** | 1 (10.0) | 7 (70.0) | **0.0198** | 0 (0.0) | 1 (4.2) | 0.9999 |
| **GVUS (p.R118C, p.S126G, p.A143T), n (%)** | 5 (50.0) | 0 (0.0) | **0.0325** | 1 (4.2) | 3 (12.5) | 0.6085 |
| ACR: albumin/creatinine-ratio, CKD: chronic kidney disease, DBP: diastolic blood pressure, DS3: Disease Severity Scoring System, eGFR: estimated glomerular filtration rate, ERT: enzyme replacement therapy, FD: Fabry disease, GVUS: genetic variant of unknown significance, ICD: implantable cardioverter device, IVS: interventricular septum thickness, Ktx: kidney transplantation, LVH: left ventricular hypertrophy, defined as LVMi > references (males: >115 g/m^2^ and females:>95 g/m^2^), LVEF: left ventricular ejection fraction, LVMi: left ventricular mass index, lyso-Gb_3_: globotriaosylsphingosine with an upper limit of normal of 1.8 ng/ml, MSSI: Mainz Severity Score Index, RAAS: renin-angiotensin-aldosterone-system, SBP: systolic blood pressure. Albuminuria: excretion rate of albumin in the urine ≥30 mg/g creatinine; CKD stage G1 (≥90 ml/min/1.73 m^2^), G2 (60-89 ml/min/1.73 m^2^), G3 (30-59 ml/min/1.73 m^2^). | | | | | | |

| Supplemental Table 2: Comparison of baseline values of cardiac parameters between females and males. | | | | | | |
| --- | --- | --- | --- | --- | --- | --- |
| **group** | **migalastat-treated** | | | **ERT-treated** | | |
|  | **females [n=10]** | **males [n=10]** | **p-value** | **females [n=24]** | **males [n=24]** | **p-value** |
| **LVM, g** | 164.6 [99.7 to 266.9] | 283 [158 to 444] | **0.0101** | 196.1 [109.7 to 472.7] | 272.3 [155.4 to 17.7] | 0.0857 |
| **LVMi, g/m²** | 88.2 [48.6 to 151.3] | 138 [76 to 220] | 0.1088 | 114.4 [64.9 to 297.8] | 130.2 [80.1 to 396.0] | 0.2438 |
| **LVEDd, mm** | 41.0 [31.0 to 47.0] | 49.0 [37.0 to 53.0] | **0.0361** | 40.5 [36.0 to 51.0] | 47.0 [30.0 to 56.0] | **0.0028** |
| **LVESd, mm** | 27.0 [20.0 to 35.0] | 30.0 [19.0 to 39.0] | 0.3874 | 30.0 [17.0 to 38.0] | 32.5 [20.0 to 46.0] | 0.1599 |
| **IVS, mm** | 12.5 [9.0 to 17.0] | 15.0 [11.0 to 27.0] | 0.2990 | 14.0 [7.0 to 22.0] | 14.5 [10.0 to 30.0] | 0.7267 |
| **PW, mm** | 11.5 [7.0 to 15.0] | 13.0 [7.0 to 25.0] | 0.2309 | 12.5 [8.0 to 20.0] | 13.0 [8.0 to 25.0] | 0.7057 |
| **RVDd, mm** | 27.0 [24.0 to 31.0] | 28.0 [19.0 to 39.0] | 0.9999 | 28.0 [20.0 to 36.0] | 30.0 [19.0 to 36.0] | 0.3129 |
| **LVEF, %** | 63.0 [51.6 to 74.6] | 54.4 [33.9 to 75.6] | 0.2224 | 60.0 [50.0 to 67.0] | 62.8 [33.7 to 71.0] | 0.1172 |
| **LA diameter, mm** | 33 [24 to 40] | 38 [25 to 48] | **0.0454** | 35.2 [24.0 to 45.0] | 34.0 [24.0 to 55.0] | 0.9825 |
| **LA volume, ml** | 33 [28 to 93] | 52 [42 to 120] | **0.0103** | 46 [18 to 77] | 50 [30 to 113] | 0.0909 |
| **LA volume index, ml/m²** | 19 [14 to55] | 25 [20 to 57] | **0.0418** | 23 [11 to36] | 27 [14 to 58] | **0.0557** |
| **E-wave, m/s** | 0.63 [0.46 to 0.98] | 0.62 [0.44 to 1.40] | 0.9999 | 0.74 [0.46 to 0.97] | 0.75 [0.45 to 1.10] | 0.5575 |
| **A-wave, m/s** | 0.74 [0.40 to 01.00] | 0.48 [0.34 to 5.0] | 0.1812 | 0.62 [0.31 to 0.81] | 0.57 [0.27 to 0.90] | 0.3538 |
| **E/A** | 0.78 [0.68 to 1.57] | 1.17 [0.12 to 2.56] | 0.4908 | 1.30 [0.65 to 2.10] | 1.35 [0.67 to 2.82] | 0.9720 |
| **Deceleration velocity, ms** | 144 [115 to 265] | 155 [72 to 240] | 0.7251 | 199 [115 to 290] | 212 [108 to 362] | 0.5664 |
| **E‘-lateral** | 0.07 [0.06 to 0.10] | 0.07 [0.05 to 0.13] | 0.9727 | 0.11 [0.05 to 0.20] | 0.12 [0.05 to 0.24] | 0.3735 |
| **E/E‘-lateral** | 8.7 [4.6 to 10.7] | 7.2 [4.6 to 23.3] | 0.9273 | 6.7 [3.9 to 13.5] | 5.5 [2.8 to 15.6] | 0.3338 |
| **LaSct, %** | -15.8 [-24.2 to 1.2] | -7.8 [-22.4 to 0.57] | 0.2973 | -11.1 [-27.7 to -2.4] | -10.0 [-24.0 to -4.6] | 0.9490 |
| **LaScd, %** | -19.2 [-32.8 to -2.72] | -8.5 [-28.0 to -3.8] | 0.3865 | -20.4 [-45.3 to -2.4] | -25.5 [-41.8 to -6.1] | 0.4742 |
| **LaSr, %** | 40.7 [12.1 to 50.0] | 20.1 [7.0 to 47.2] | 0.2224 | 32.0 [7.6 to 53.3] | 35.6 [12.9 to 59.5] | 0.6461 |
| **GLS, %** | -14.7 [-19.1 to -8.3] | -10.6 [18.8 to -5.7] | 0.3734 | -16.7 [-19.6 to -5.3] | -13.9 [-23.0 to -6.5] | 0.3196 |
| **RVFWSL, %** | -18.7 [-41.2 to -0.4] | -16.2 [-29.9 to -7.0] | 0.7962 | -23.9 [-30.7 to -3.1] | -19.0 [-27.8 to -8.1] | 0.5065 |
| **RV lateral diameter, mm** | 5 [4 to 7] | 7 [4 to 9] | **0.0380** | 5 [3 to 8] | 6 [4 to 11] | 0.1860 |
| BSA: body surface area, LVM: left ventricular mass, LVMi: left ventricular mass index, LVEDd: left ventricular enddiastolic diameter, LVESd: left ventricular endsystolic diameter, IVS: interventricular septum thickness, PW: posterior wall thickness, RVDd: right ventricular diastolic diameter, LVEF: left ventricular ejection fraction, LaSct: left atrial contraction “booster” strain, LaScd: left atrial conduit strain, LaSr: left atrial reservoir strain, GLS: global longitudinal strain average, RVFWSL: right ventricular free wall longitudinal strain. | | | | | | |

| Supplemental Table 3: Comparison of outcomes for cardiac parameters between females and males. | | | | | | |
| --- | --- | --- | --- | --- | --- | --- |
| **group** | **migalastat-treated** | | | **ERT-treated** | | |
|  | **females [n=10]** | **males [n=10]** | **p-value** | **females [n=24]** | **males [n=24]** | **p-value** |
| **Parameter (change per year)** |  |  |  |  |  |  |
| **LVM, g** | 5.96 [-62.8 to 16.4] | -14.00 [-127.8 to 68.8] | 0.2799 | 1.90 [-44.3 to 24.6] | 0.73 [-36.5 to 26.4] | 0.8785 |
| **LVMi, g/m²** | 2.57 [-36.8 to 10.0] | -7.2 [-14.2 to 27.9] | 0.2370 | 0.86 [-10.9 to 13.9] | -1.17 [-15.6 to 15.7] | 0.3523 |
| **LVEDd, mm** | 0.47 [-7.6 to 5.1] | -1.44 [-13.7 to 3.6] | 0.3246 | 0.0 [-3.9 to 3.5] | -0.27 [-4.0 to 2.1] | 0.4805 |
| **LVESd, mm** | 1.03 [-5.6 to 3.3] | -2.2 [-9.3 to 3.7] | 0.1903 | 0.0 [-1.6 to 9.2] | 0.03 [-5.5 to 9.9] | 0.5692 |
| **IVS, mm** | -0.29 [-1.2 to 1.6] | 0.00 [-5.1 to 1.0] | 0.6820 | 0.0 [-0.6 to 2.1] | 0.11 [-1.7 to 3.5] | 0.4432 |
| **PW, mm** | 0.0 [-1.1 to 2.4] | 0.00 [-8.6 to 1.6] | 0.9704 | 0.0 [-2.3 to 2.1] | 0.11 [-1.8 to 1.5] | 0.3068 |
| **RVDd, mm** | 0.46 [-3.1 to 3.3] | 1.45 [-9.9 to 7.1] | 0.2100 | -0.48 [-5.2 to 1.5] | 0.29 [-1.5 to 1.7] | **0.0277** |
| **LVEF, %** | -0.29 [-8.5 to 15.3] | 0.9 [-18.4 to 8.9] | 0.7802 | 0.1 [-3.5 to 26.8] | 0.77 [-7.6 to 6.2] | 0.7660 |
| **LA diameter, mm** | 0.70 [-1.8 to 7.2] | 0.50 [-2.4 to 8.8] | 0.7802 | 0.0 [-2.1 to 3.5] | -0.11 [-5.3 to 1.9] | 0.8064 |
| **LaSct, %** | 0.98 [-2.45 to 11.77] | 0.87 [-11.88 to 7.10] | 0.9118 | 0.85 [-7.19 to 7.95] | 0.28 [-3.51 to 4.81] | 0.4604 |
| **LaScd, %** | 0.87 [-11.1 to 17.6] | -0.81 [-10.11 to 4.49] | 0.1655 | 0.10 [-6.56 to 25.58] | 0.18 [-13.91 to 3.93] | 0.8001 |
| **LaSr, %** | -1.08 [-20.7 to 2.23] | 0.20 [-11.59 to 19.26] | **0.0433** | -0.96 [-31.95 to 12.68] | -0.26 [-6.17 to 9.92] | 0.4701 |
| **LA volume index, ml/m²** | 1.95 [-4.43 to 5.16] | 1.76 [-1.24 to 4.48] | 0.6334 | 0.89 [-2.58 to 4.56] | -0.64 [-16.8 to 11.28] | 0.0621 |
| **E wave, m/s** | 0.03 [-0.1 to 0.1] | 0.00 [-0.1 to 0.2] | 0.7962 | -0.03 [-0.1 to 0.0] | -0.01 [-0.1 to 0.1] | 0.0772 |
| **A wave, m/s** | 0.06 [-0.2 to 0.3] | -0.02 [-1.5 to 0.1] | 0.1206 | 0.0 [-0.1 to 0.1] | 0.0 [-0.1 to 0.1] | 0.6306 |
| **E/A** | -0.03 [-0.4 to 0.4] | 0.14 [-0.3 to 0.5] | 0.3357 | -0.03 [-0.1 to 4.] | 0.0 [-0.4 to 0.4] | 0.3155 |
| **Deceleration velocity, ms** | 15.1 [-44.0 to 63.0] | 0.6 [-36.0 to 68.6] | 0.7430 | -0.6 [-53.3 to 32.3] | -0.83 [-40.4 to 61.5] | 0.8050 |
| **E’-lateral** | 0.00 [0.0 to 0.01] | 0.01 [-0.02 to 0.02] | 0.9999 | 0.0 [0.0 to 0.0] | 0.0 [-0.03 to 0.01] | 0.4532 |
| **GLS, %** | 0.07 [-2.9 to 1.7] | 1.38 [-6.6 to 13.3] | 0.1615 | 0.85 [-0.9 to 6.6] | 0.37 [-20.7 to 3.6] | 0.3314 |
| **RVFWSL, %** | -1.5 [-8.6 to 25.7] | -1.3 [11.0 to 5.7] | 0.7962 | -0.3 [-7.3 to 3.0] | 0.4 [-4.3 to 6.3] | 0.3101 |
| BSA: body surface area, LVM: left ventricular mass, LVMi: left ventricular mass index, LVEDd: left ventricular enddiastolic diameter, LVESd: left ventricular endsystolic diameter, IVS: interventricular septum thickness, PW: posterior wall thickness, RVDd: right ventricular diastolic diameter, LVEF: left ventricular ejection fraction, LaSct: left atrial contraction “booster” strain, LaScd: left atrial conduit strain, LaSr: left atrial reservoir strain, GLS: global longitudinal strain average, RVFWSL: right ventricular free wall longitudinal strain. | | | | | | |

| Supplemental table 4: Correlation between NT-proBNP, ACR and lyso-Gb_3_ with cardiac parameters at baseline. | | | | | | |
| --- | --- | --- | --- | --- | --- | --- |
|  | **ERT** | | | **Migalastat** | | |
|  | **Slope [95% CI]** | **R²** | **p-value** | **Slope [95% CI]** | **R²** | **p-value** |
| **NT-proBNP** |  |  |  |  |  |  |
| **LVMI** | -0.0019 to 0.0301 | 0.1076 | 0.0823 | 0.0057 to 0.0783 | 0.2882 | **0.0263** |
| **eGFR** | -0.0132 to -0.0037 | 0.3185 | **0.0012** | -0.0314 to -0.0009 | 0.2166 | **0.0387** |
| **LaSct** | -0.0017 to 0.0028 | 0.0242 | 0.6118 | 0.0037 to 0.015 | 0.4281 | **0.0032** |
| **LaScd** | -0.0018 to 0.0034 | 0.0423 | 0.5199 | 0.0037 to 0.0190 | 0.3821 | **0.0063** |
| **LaSr** | -0.0055 to 0.0028 | 0.0456 | 0.4836 | -0.0301 to -0.0118 | 0.5969 | **0.0002** |
| **GLS** | -0.0011 to 0.0016 | 0.0298 | 0.6332 | 0.0041 to 0.0094 | 0.6479 | **0.0001** |
| **RVFWSL** | -0.0027 to 0.0011 | 0.0717 | 0.3765 | 0.0013 to 0.0169 | 0.2788 | **0.0243** |
| **ACR** |  |  |  |  |  |  |
| **LVMI** | -0.0154 to 0.0472 | 0.0271 | 0.3101 | 0.0047 to 0.0623 | 0.3491 | **0.0261** |
| **eGFR** | -0.0225 to -0.0020 | 0.1296 | **0.0208** | -0.0192 to 0.0098 | 0.0336 | 0.4966 |
| **LaSct** | -0.0013 to 0.0032 | 0.0371 | 0.4031 | -0.0063 to 0.0046 | 0.0096 | 0.7384 |
| **LaScd** | -0.0073 to 0.0047 | 0.0112 | 0.6475 | -0.0035 to 0.0117 | 0.1031 | 0.2629 |
| **LaSr** | -0.0070 to 0.0078 | 0.0007 | 0.9103 | -0.014 to 0.0075 | 0.0348 | 0.5232 |
| **GLS** | -0.0023 to 0.0024 | 0.0002 | 0.9518 | -0.0024 to 0.0044 | 0.0331 | 0.5337 |
| **RVFWSL** | -0.0042 to 0.0033 | 0.0028 | 0.8205 | -0.0043 to 0.0090 | 0.0469 | 0.4569 |
| **lyso-Gb_3_** |  |  |  |  |  |  |
| **LVMI** | -0.5700 to 0.4547 | 0.0021 | 0.8187 | 4.6120 to 12.2800 | 0.5951 | **0.0003** |
| **eGFR** | -0.0864 to 0.2365 | 0.0339 | 0.3481 | -3.201 to 0.0686 | 0.1938 | 0.0593 |
| **LaSct** | -0.043 to 0.1013 | 0.0843 | 0.3863 | -0.8076 to 1.4430 | 0.0236 | 0.5564 |
| **LaScd** | -0.160 to 0.0433 | 0.157 | 0.2271 | -0.1283 to 2.4390 | 0.1970 | 0.0743 |
| **LaSr** | -0.1395 to 0.1976 | 0.0166 | 0.7054 | -3.4780 to 0.5317 | 0.1405 | 0.1382 |
| **GLS** | -0.095 to 0.0444 | 0.1158 | 0.4095 | 0.0115 to 1.1240 | 0.2398 | **0.0460** |
| **RVFWSL** | -0.0486 to 0.1067 | 0.0736 | 0.4197 | -0.4992 to 2.1090 | 0.1035 | 0.2080 |
| ACR: albumin-creatinine ratio, eGFR: estimated glomerular filtration rate, lyso-Gb_3_: globotriaosylsphingosine, LVMi: left ventricular mass index, LaSct: left atrial contraction “booster” strain, LaScd: left atrial conduit strain, LaSr: left atrial reservoir strain, GLS: global longitudinal strain average, RVFWSL: right ventricular free wall longitudinal strain. | | | | | | |

# Supplementary Figures

| 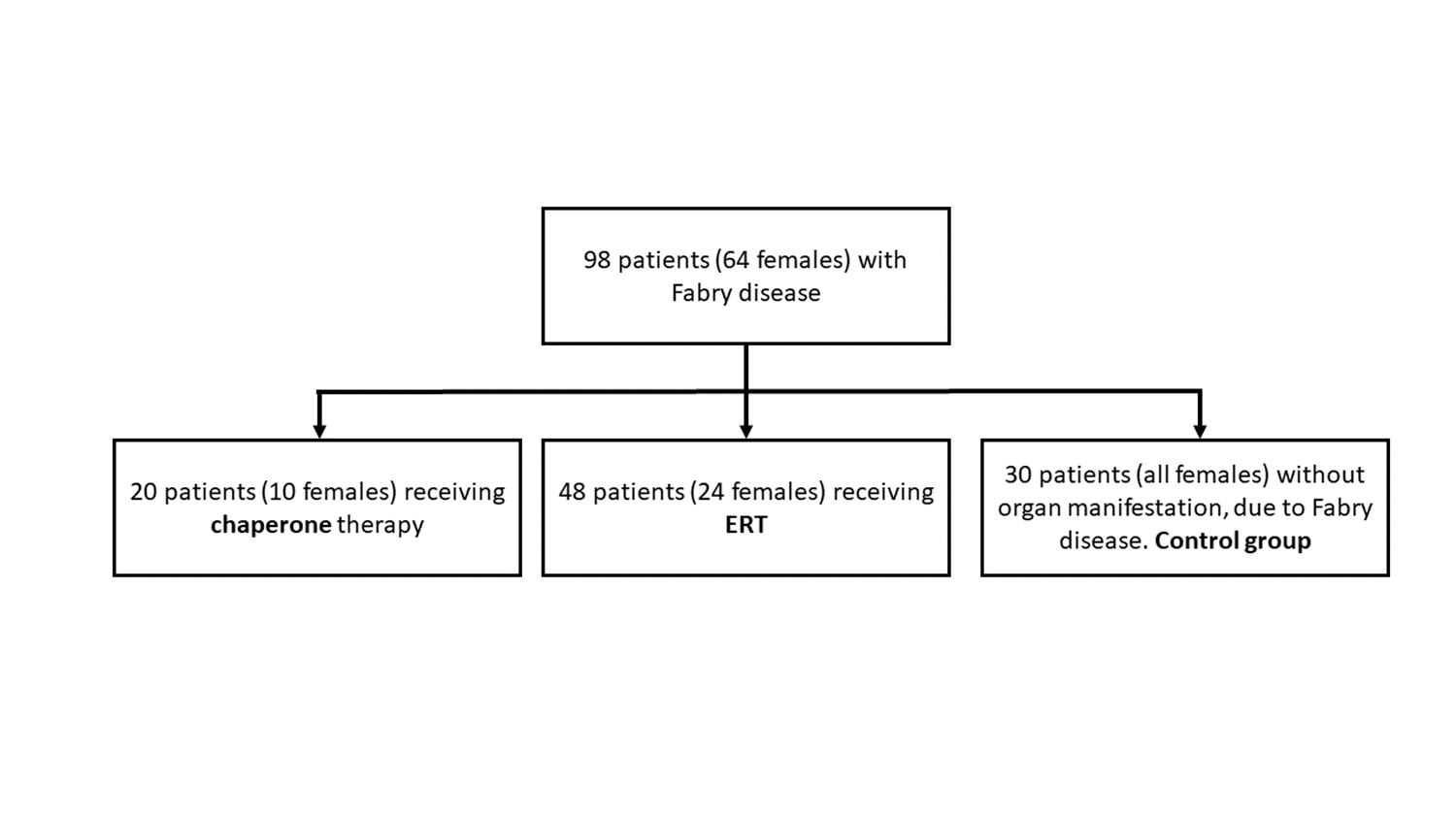 |
| --- |
| **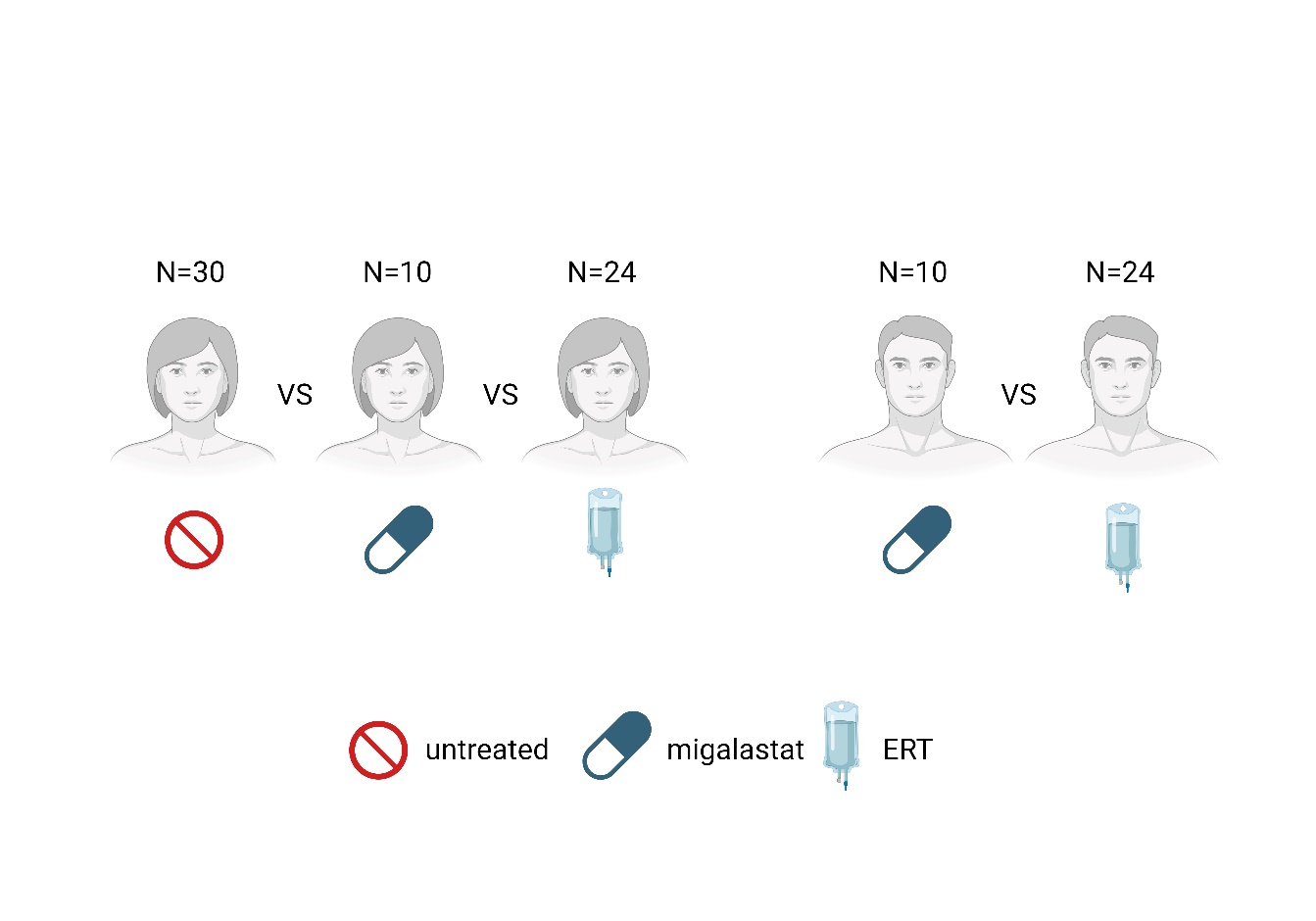** |
| Supplemental Figure 1: Overview of the study population and their analyses. ERT: enzyme replacement therapy. |

| 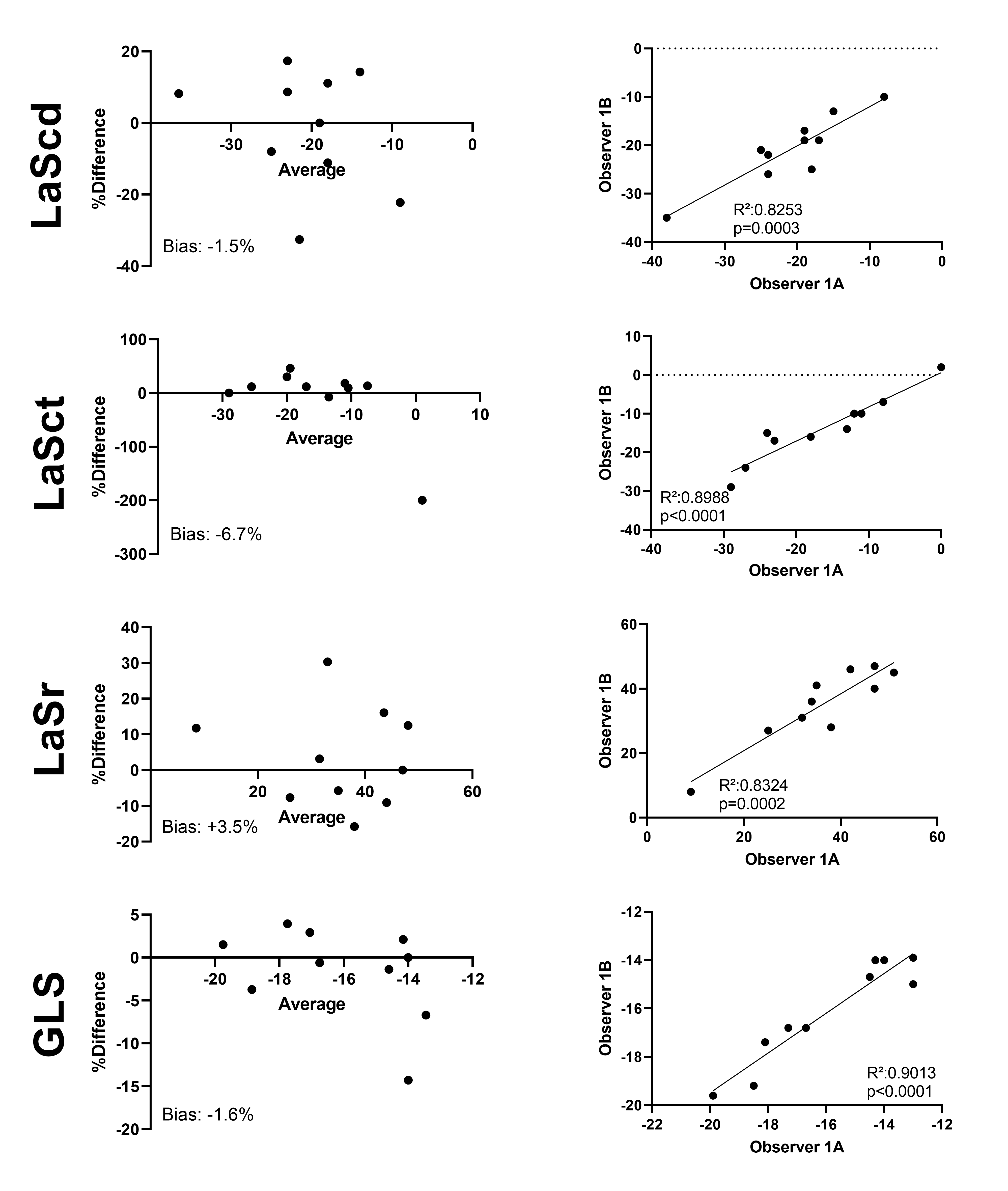 |
| --- |
| Supplemental Figure 2: Intraobserver variability LaSct: left atrial contraction “booster” strain, LaScd: left atrial conduit strain, LaSr: left atrial reservoir strain, GLS: global longitudinal strain average; R^2^: coefficient of determination, R^2^= 0.0-0.3: low; R^2^=0.3-0.5: moderate; R^2^=>0.5: high. |
| 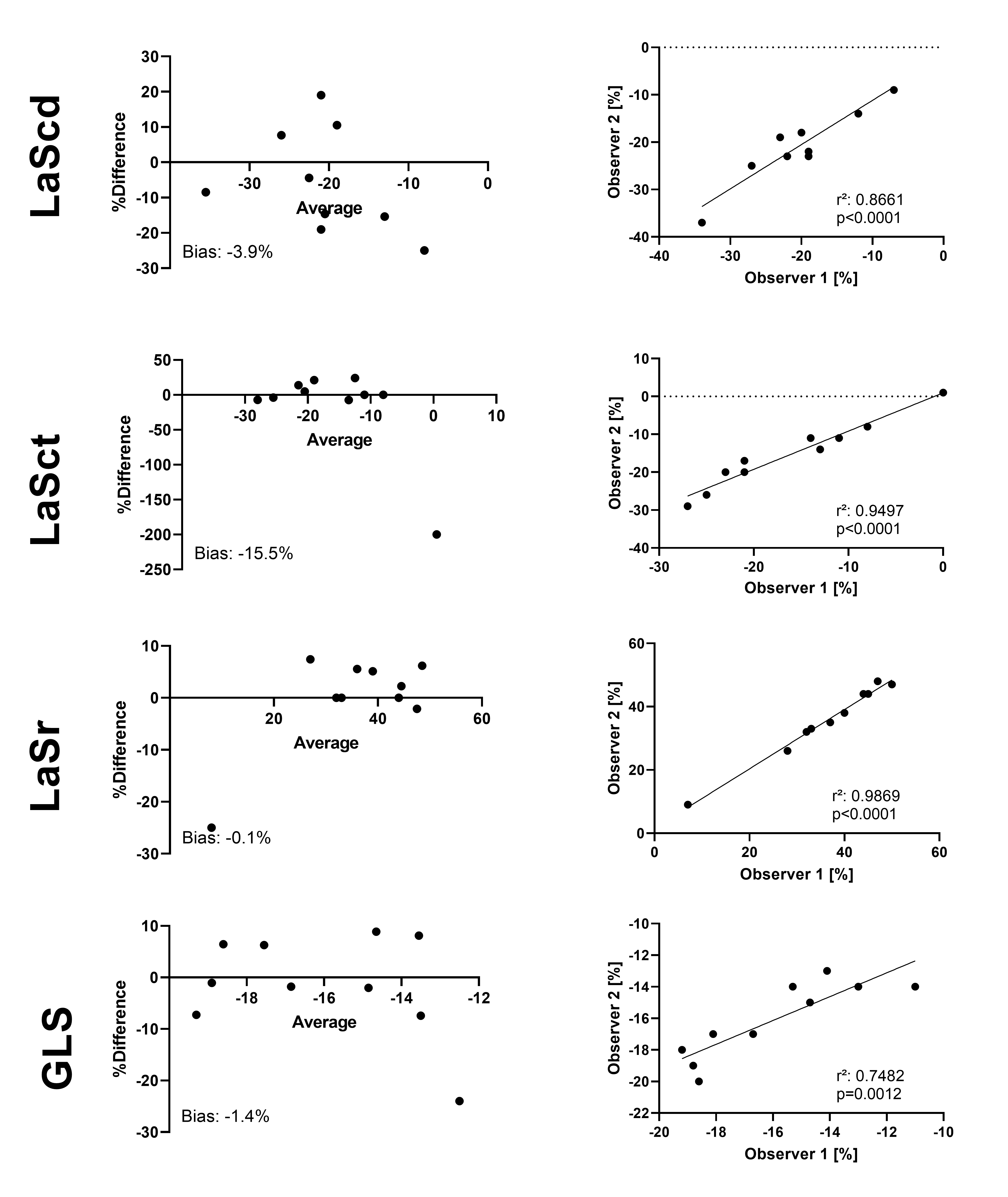 |
| Supplemental Figure 3: Interobserver variability LaSct: left atrial contraction “booster” strain, LaScd: left atrial conduit strain, LaSr: left atrial reservoir strain, GLS: global longitudinal strain average; R^2^: coefficient of determination, R^2^= 0.0-0.3: low; R^2^=0.3-0.5: moderate; R^2^=>0.5: high. |

|  |
| --- |
| Supplemental Figure 4: Impact of LVH at baseline on left ventricular mass index. Yearly changes were analyzed by one sample Wilcoxon test.  *****p<0.05 |

| 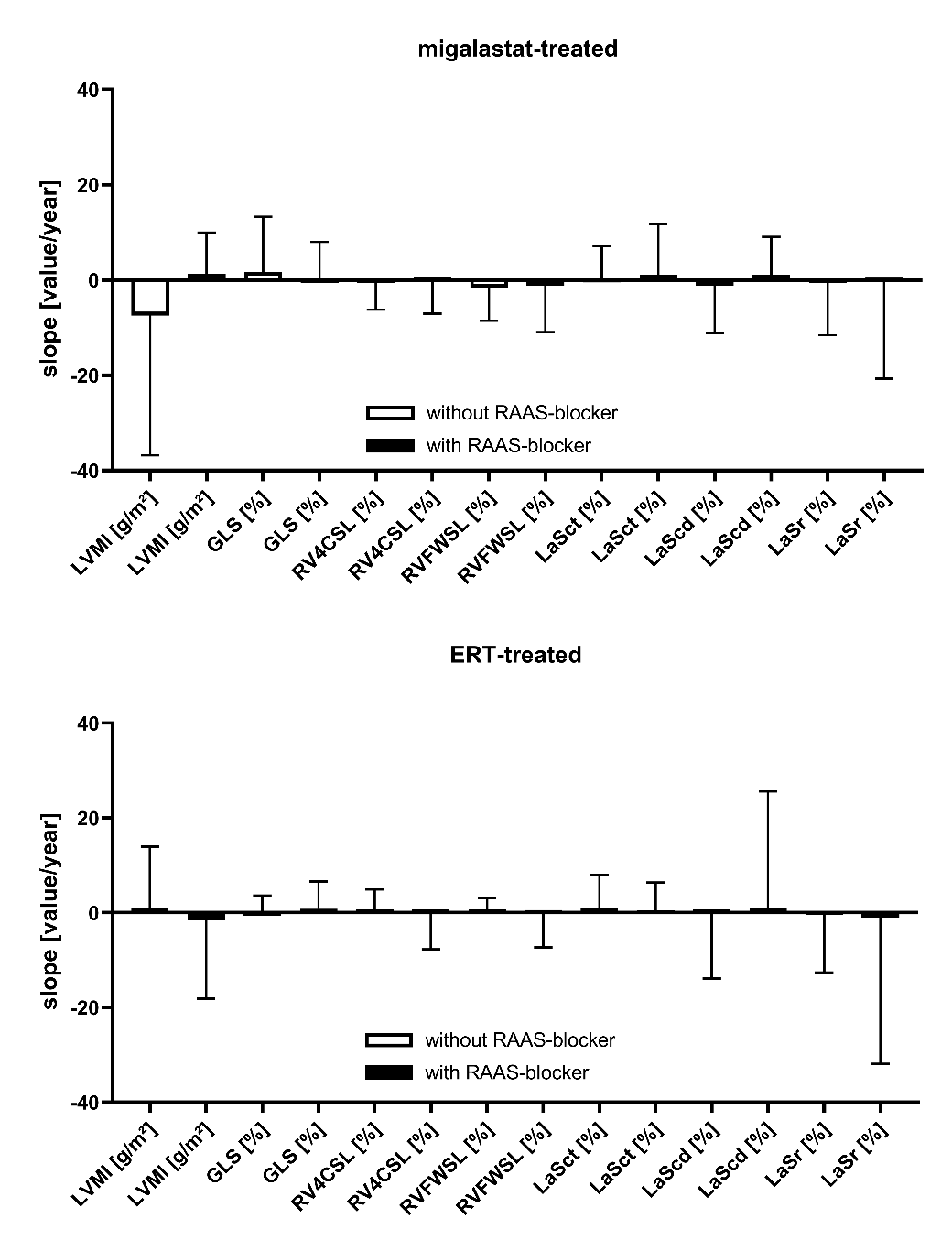 |
| --- |
| Supplemental Figure 5: Effects of RAAS-blockers on cardiovascular parameters Left without RAAS-Blocker – right with RAAS-blocker; LVMi: left ventricular mass index, GLS: global longitudinal strain average, LaSct: left atrial contraction “booster” strain, LaScd: left atrial conduit strain, LaSr: left atrial reservoir strain, RV4CSL: right ventricular 4-chamber strain, RVFWSL: right ventricular free wall longitudinal strain, RAAS: renin-angiotensin-aldosterone-system, ERT: enzyme replacement therapy. Mann-Whitney U Test; one sample Wilcoxon Test, *****p<0.05 |
